# Cumulus Cell Transcriptome after Cumulus-Oocyte Complex Exposure to Nanomolar Cadmium in an In Vitro Animal Model of Prepubertal and Adult Age

**DOI:** 10.3390/biology12020249

**Published:** 2023-02-04

**Authors:** Nicola Antonio Martino, Ernesto Picardi, Elena Ciani, Anna Maria D’Erchia, Luisa Bogliolo, Federica Ariu, Antonella Mastrorocco, Letizia Temerario, Luigi Mansi, Valeria Palumbo, Graziano Pesole, Maria Elena Dell’Aquila

**Affiliations:** 1 Department of Biosciences, Biotechnologies & Environment, University of Bari Aldo Moro, Via Edoardo Orabona, 70125 Bari, Italy; 2 Department of Veterinary Medicine, University of Sassari, Via Vienna n. 2, 07100 Sassari, Italy

**Keywords:** adult sheep, prepubertal lamb, cumulus cell, transcriptome, RNA-sequencing, cadmium, endocrine disrupting chemicals, female reproductive toxicity, non-invasive biomarker, oocyte competence

## Abstract

**Simple Summary:**

Cadmium (Cd), a highly toxic environmental contaminant, negatively affects human and animal fertility in females. In sheep, Cd displays age-dependent bioaccumulation at ovarian level. At environmental nanomolar concentrations, it reduces oocyte fertilization by inducing oxidative stress on cumulus cells (CCs), a cell population of the cumulus-oocyte complex (COC) supporting oocyte growth, functional maturation and fertilization. In this study, the modifications induced by Cd exposure to all the genes expressed in CCs of in vitro matured COCs, recovered from the ovaries of adult and prepubertal sheep, were analysed by RNA sequencing. A set of genes significantly dysregulated upon Cd exposure was identified. Effects of Cd were more relevant in CCs from adult than from prepubertal COCs. Most genes were upregulated while a minority of them were downregulated. Some genes were already known as involved in ovarian activity or Cd-induced effects, whereas others were completely new in these fields. These findings identify in the sheep, an important livestock species with translational relevance in human reproduction, the set of genes controlling oocyte functional competence, altered by Cd. These biomarkers will make it possible to identify oocytes that cannot be fertilized to evaluate whether they are to be discarded or recovered with detoxifying treatments.

**Abstract:**

Cadmium (Cd), a highly toxic pollutant, impairs oocyte fertilization, through oxidative damage on cumulus cells (CCs). This study analysed the transcriptomic profile of CCs of cumulus-oocyte complexes (COCs) from adult and prepubertal sheep, exposed to Cd nanomolar concentration during in vitro maturation. In both age-groups, CCs of matured oocytes underwent RNA-seq, data analysis and validation. Differentially expressed genes (DEGs) were identified in adult (*n* = 99 DEGs) and prepubertal (*n* = 18 DEGs) CCs upon Cd exposure. Transcriptomes of adult CCs clustered separately between Cd-exposed and control samples, whereas prepubertal ones did not as observed by Principal Component Analysis. The transcriptomic signature of Cd-induced CC toxicity was identified by gene annotation and literature search. Genes associated with previous studies on ovarian functions and/or Cd effects were confirmed and new genes were identified, thus implementing the knowledge on their involvement in such processes. Enrichment and validation analysis showed that, in adult CCs, Cd acted as endocrine disruptor on DEGs involved in hormone biosynthesis, cumulus expansion, regulation of cell signalling, growth and differentiation and oocyte maturation, whereas in prepubertal CCs, Cd affected DEGs involved in CC development and viability and CC-oocyte communications. In conclusion, these DEGs could be used as valuable non-invasive biomarkers for oocyte competence.

## 1. Introduction

In recent years, a consistent body of studies reported that environmental contamination, due to chemicals of industrial and biological origin, affects female fertility in humans and animals [1,2]. Heavy metals are a major concern for reproductive health, due to their high global annual emission rate [3] and among them, Cadmium (Cd) is one of the most toxic.

In in vivo studies, in laboratory animal models, multiple Cd effects have been reported, such as interference with the hypothalamic–pituitary–ovarian axis, reduced steroidogenesis, inhibition of follicle and oocyte development, impaired ovulation and oocyte pick-up by the tubal epithelium, delayed embryo development and implantation, restricted foetal growth and pregnancy complications [4,5,6,7,8,9,10,11]. Cadmium has been detected in human follicular fluids [12,13,14,15] and animal ovarian tissues [16]. In sheep, it was shown as a trace element with a very high age-dependent ovarian bioaccumulation [16].

In vitro exposure to Cd was reported to negatively affect oocyte maturation, subsequent fertilization and embryo development in different animal species [16,17,18,19,20,21,22,23] with most of the studies performed by analysing the effects of micromolar Cd concentrations. In a previous study in a sheep model, we found that environmental nanomolar Cd concentrations did not compromise oocyte nuclear maturation; rather, it adversely affected oocyte developmental competence. Indeed, exposure to nanomolar Cd concentrations during in vitro maturation (IVM) impaired oocyte in vitro fertilization by inducing oxidative stress, observed as increased ROS (reactive oxygen species) levels and lipid peroxidation, prevailingly on cumulus cells (CCs) rather than on the oocyte [16]. Based on these data, it emerged that nanomolar Cd induces essentially functional effects while the cumulus–oocyte complex (COC) morphology remains unaltered. Furthermore, high percentages of oocytes exposed to Cd reach meiotic maturation. These observations prompted us to conduct studies aimed at identifying non-invasive biomarkers of nanomolar Cd exposure in CCs, allowing oocyte preservation for clinical use in IVF or ICSI programs followed by embryo culture and transfer.

Cumulus cells are a follicular granulosa cell subpopulation which represent an ideal cell substrate for the identification of non-invasive biomarkers of oocyte quality. These somatic cells surround growing oocytes, to which they are directly connected through cytoplasmic protrusions, and can be isolated from the COC without compromising oocyte viability. CCs support oocyte growth, maturation and acquisition of developmental competence [24,25,26,27,28]. Indeed, oocyte nuclear and cytoplasmic maturation depends on rapid transcriptional events, governed by paracrine and autocrine signalling before ovulation in which CCs play significant roles [28,29,30,31]. Once matured, the metaphase II (MII) oocyte is less transcriptionally active and relies on stored mRNA transcripts, acquired throughout maturation, to undergo successful fertilization and early embryo development until embryonic genome activation (EGA) [32,33,34]. Moreover, other than cytoplasmic stored transcripts, CCs provide additional transcripts to the oocyte by active transportation through trans-zonal projections [35,36]. This is why investigating transcriptional CC activity is a key strategy for detecting biomarkers of oocyte quality, improving the outcomes of assisted reproductive programs.

Studies on CCs transcriptomic profile have started in recent years and have developed rapidly in human as well as in some animal species, initially using microarrays [37] and then by RNA-seq [27]. In reproductive toxicology [38], this technology can open the way to the identification of new biomarkers of oocyte functional damage induced by specific contaminants. The aim of the present study was to perform CC transcriptome analysis for the identification of non-invasive biomarkers of oocyte functional impairment due to in vitro exposure to Cd at environmental nanomolar concentration. The IVM of sheep oocytes was used as a large animal in vitro model, with oocytes recovered from adult sheep or prepubertal lambs, representing those with high or low developmental competence, respectively.

## 2. Materials and Methods

### 2.1. Chemicals

All chemicals for in vitro cultures and analyses were purchased from Sigma-Aldrich (Milan, Italy) unless otherwise indicated.

### 2.2. Collection of Ovaries

Ovaries were collected from prepubertal lambs (under 2 months old) and adult sheep (3–5 years old) at a local slaughterhouse (Fin. Sud Import s.r.l.; Conversano, Bari). All animals were subjected to routine veterinary inspection in accordance with the specific health requirements stated in Council Directive 89/556/ECC and subsequent modifications. Ovaries were transported to the laboratory at room temperature within 2 to 4 h from slaughter.

### 2.3. In Vitro Maturation (IVM)

For the retrieval of cumulus–oocyte complexes (COCs), ovaries were processed differently in relation to the donor age. Ovaries of prepubertal lambs underwent the slicing procedure [39], whereas ovaries from adult sheep underwent follicular fluid aspiration from large developing follicles using a 18G needle. In both procedures, the follicular contents were released in sterile Petri dishes containing phosphate-buffered saline (PBS) supplemented with 1 mg/mL heparin. Only COCs with several intact cumulus cells layers and homogenous cytoplasm were selected for culture. In vitro maturation was performed as previously reported [40]. Briefly, IVM culture medium was composed by TCM-199 with Earle’s salts, buffered with 5.87 mM 4-(2-hydroxyethyl)-1- piperazine ethane sulfonic acid (HEPES) and 33.09 mM sodium bicarbonate and supplemented with 0.1 g/L L-glutamine, 2.27 mM sodium pyruvate, calcium-l-lactate pentahydrate (1.62 mM calcium and 3.9 mM Lactate, 50 μg/mL gentamicin, 20% (*v*/*v*) Foetal Calf Serum (FCS), 10 μg/mL ovine follicle stimulating hormone (FSH) and 20 μg/mL ovine luteinizing hormone (LH), and 1 μg/mL 17 beta oestradiol. Before IVM, collected COCs were washed three times in TCM-199 with Hank’s salts (Gibco^®^, Life Technologies, Paisley, UK) supplemented with 10% FCS. COCs were individually cultured in single 10 μL microdrops of IVM medium placed in 60 mm petri dishes covered with pre-equilibrated paraffin oil. This individual IVM culture system allows separation of CCs from MII oocytes from those of immature ones. IVM culture was performed for 24 h at 38.5 °C under 5% CO_2_ in air. COCs were exposed to Cd at 100 or 0 (controls) nM. This concentration was chosen on the basis of previous studies reporting that 100 nM CdCl_2_ significantly reduced the fertilization rates of oocytes from prepubertal and adult sheep [16]. Cadmium working solution was prepared on the day of use, starting from a 10 mM CdCl_2_ stock solution in distilled water.

### 2.4. Cumulus Cells Isolation from Matured Oocytes

After IVM, individual COCs underwent CC removal and oocyte meiotic stage assessment by polar body (PB) visualization under a Nikon SMZ-1500 stereomicroscope. For each experimental condition (100 nM CdCl_2_ and controls), CCs isolated from COCs with matured oocytes, showing the 1st PB extruded, were pooled in groups of 20–25 cumuli/group, collected in RNAse-free tubes and washed twice in cold PBS by centrifugation at 300× *g* for 1 min. After supernatant removal, CC pellets were snap-frozen in liquid nitrogen and stored at −80°C until molecular analyses. The meiotic stage of oocytes which did not show the 1st PB extruded was assessed by nuclear chromatin evaluation.

### 2.5. Oocyte Nuclear Chromatin Evaluation by Epifluorescence Microscopy

To evaluate nuclear chromatin, oocytes were fixed in 3.8% formaldehyde solution in PBS, stained with 2.5 μg/mL Hoechst 33258 in 3:1 (*v*/*v*) glycerol/PBS, mounted on microscope slides covered with cover slips, sealed with nail polish and kept at 4 °C in the dark until observation. Oocytes were evaluated under an epifluorescence microscope (Nikon Eclipse 600, Nikon Instruments, Firenze, Italy; 400× magnification) equipped with a 346 nm excitation/460 nm emission filter, as germinal vesicle (GV), metaphase to telophase I (MI to TI), MII with 1st PB extruded and abnormal [39,41].

### 2.6. RNA-Seq and Data Analysis

For transcriptomic analysis, for each experimental condition (prepubertal Cd-treated, prepubertal controls, adult Cd-treated and adult controls), 5 groups of CCs from matured COCs were used. In each group, CCs isolated from 20–25 matured oocytes were pooled and processed for RNA extraction and CCs from equal numbers of treated and control COCs were analysed. Total RNA was extracted and purified with the mirVana kit (Thermo Fisher Scientific, Waltham, MA, USA) following the manufacturer’s protocol. During extraction procedures, RNAs were treated with DNase to exclude any contamination of genomic DNA. Total RNA integrity was evaluated using the 2100-Bioanalyzer (Agilent Technologies, Santa Clara, California, USA) with the RNA PicoLab Chip (Agilent Technologies) and RNA concentration and purity were evaluated using NanoDrop 2000c (Thermo Fisher Scientific). RNA-seq libraries were prepared using the Illumina’s TruSeq Stranded Total RNA Sample Preparation Kit (Illumina, San Diego, CA, USA), according to the manufacturer’s protocol. Sequencing was performed on Illumina NextSeq500 platform, generating 100 bp paired-end reads. Raw reads in FASTQ format were quality checked using the FastQC program (http://www.bioinformatics.babraham.ac.uk/projects/fastqc, accessed on 12 December 2022) and adaptors as well as low-quality regions (phred cutoff of 25) were trimmed using Trim_Galore. Cleaned reads were aligned onto the reference genome of the ovine species (Oar_v3.1 from UCSC) using STAR (version 020201) [42] with default parameters. Read counts per gene were performed by FeatureCounts (version 1.6.0). Normalized counts of 1000 most variable genes were used to perform a PCA analysis. Differentially expressed genes (DEGs) between Cd-treated and control samples were detected by using the DESeq2 R package [43], selecting only genes with a *p* value < 0.05 and |log2fc| > 1.2, and displayed as heatmaps (using the zscore (z = (X − μ)/σ) conversion).

### 2.7. Gene Network Analysis

The GeneMANIA (http://www.genemania.org, accessed on 12 December 2022); [44] prediction server was used to analyse the DEG functions and to find neighbouring genes associated with DEGs by constructing the gene network.

### 2.8. RNA-Seq Data Validation by Quantitative RT-PCR

Total RNA was isolated with RNeasy Micro Kit (Qiagen, Hilden, Germany) following manufacturer’s instructions. During the procedure, RNA was treated with DNase I to exclude any potential genomic DNA contamination. The isolated RNA was measured using a Nanodrop spectrophotometer (Thermofisher) and used for reverse transcription-polymerase chain reaction (RT-PCR). The High-Capacity Complementary DNA (cDNA) Reverse Transcription kit (Life Technologies) was used to convert RNA to cDNA. Each RNA sample (1 μg) was added to 2 μL 10× RT buffer, 0.8 μL 25× dNTP mix, 2 μL RT random primers, 1 μL M-MLV RT, 1 μL RNase inhibitor and nuclease-free H_2_O for a total volume of 20 μL and then mixed gently and centrifuged briefly. Reaction tubes were incubated at 10 °C for 10 min, then at 37 °C for 120 min, and finally at 85 °C for 5 min. The relative quantification of the transcripts was carried out by Real-Time RT-PCR with the StepOne instrument (Applied Biosystems, Foster City, CA, USA). Specific ovine cDNAs were amplified by PCR using the primers shown in Table 1. PCR was performed in 20 μL reaction volume containing: 10 μL PowerUp SYBR Green PCR Master Mix (Applied Biosystems, 2×), 200 nM of each primer, 2 μL of diluted cDNA (1:10) and nuclease-free water up to 20 μL. Cycling parameters were: 95 °C for 2 min, 40 cycles of denaturation at 94 °C (45 s), annealing at 60 °C (45 s) and extension at 72 °C (45 s), final extension at 72 °C for 5 min. The analysis was carried out in triplicate. Data were collected by using the StepOne Software (Applied Biosystem) and relative quantification was performed by using a comparative method after determining the Ct (threshold cycle) values for the reference endogenous control (beta actin) and the target gene in each sample sets, according to the 2^−ΔΔCt^ method. Changes in mRNA expression levels were calculated after normalization to Beta actin. The program calculates the ΔCt and the ΔΔCt with the formulas below: ΔCt = Ct_Mean (beta actin) − Ct_Mean (target gene); ΔΔCt = ΔCt − ΔCt_Mean, so that the gene expression level =2^−ΔΔCt^. Changes in gene expression were reported as percentage changes relative to controls.

### 2.9. Statistical Analysis

Oocyte nuclear maturation rates were compared between treated and control groups by the Chi-square test. The evaluation of differences in gene expression between control and treated cells was performed by Student’s *t*-test. Differences with *p* < 0.05 were considered as statistically significant.

### 2.10. Gene Annotation and Literature Search

To determine the biological significance of our bioinformatic findings, DEGs were cross referenced with available datasets. DEGs were reviewed using the GeneCards database (http://www.genecards.org/, accessed on 12 December 2022) to retrieve general information on their structure and function and to correlate our bioinformatic findings with hallmark physiological and pathological processes in the ovary, when available (Entrez gene summary), and to search for information on gene expression at the mRNA (GTEx and Illumina BodyMap) and protein (Moped and ProteomicsDB) levels in the human normal ovary. Then, the PubMed literature database was used to search for previous studies assessing the expression and functional role of each gene in the ovary of human or other animal species, by associating the gene symbol with the following key words called, alternatively: cumulus cells, granulosa cells, oocyte, ovary.

## 3. Results

### 3.1. Nanomolar Cd Does Not Affect Meiotic Progression of Sheep Oocytes

A total of 742 COCs were cultured and analysed, 401 of which were from adult sheep and 341 from prepubertal lambs. In both age groups, exposure to nanomolar Cd during IVM did not affect oocyte meiotic progression and maturation, as no significant differences were identified in the percentages of oocytes found at the examined meiotic stages. Furthermore, no differences were observed in the MII rates of adult versus prepubertal sheep, also considering that prepubertal COCs used in this study were recovered from small antral follicles but displayed the oocyte diameter, number of CC layers and the ability to respond to gonadotropin in vitro stimulation, similarly to their adult counterparts. Overall, in all sample types, oocyte maturation rate reached values around 90% (87.6–95.0) (Table 2).

### 3.2. PCA Shows Relevant Cd-Induced Effects on Transcriptomic Profile of CCs from Adult Sheep

**Figure 1:**
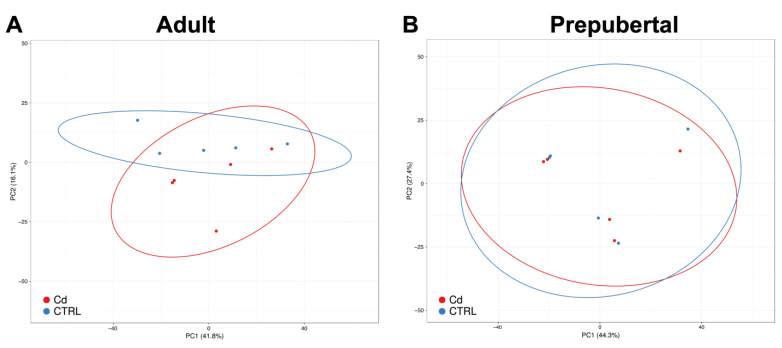
PCA of Cd-treated CCs compared with controls in adult sheep (**A**) and prepubertal lambs (**B**). Red and blue dots indicate Cd-treated and control CC samples, respectively. In adult CC samples, Cd-induced transcriptome separation along the x-axis can be seen (**A**). Instead, in CCs from prepubertal samples, the gene expression pattern is overlapping (**B**).

Transcriptomes of CCs isolated from oocytes selected after IVM as belonging to matured MII oocytes of adult sheep and prepubertal lambs were sequenced employing the Illumina technology on the NextSeq500 platform. The principal component analysis (PCA) based on gene expression data was performed by grouping sample categories according to donor age (adult versus prepubertal) and culture conditions (Cd-exposed versus controls). In adult samples, Cd exposure induced clear transcriptome separation along the x-axis (main component) (Figure 1A). This effect was not induced in CCs from prepubertal samples, in which an overlapping gene expression pattern between Cd-exposed and control samples was found (Figure 1B).

### 3.3. Cadmium Induces Higher Number of DEGs in CCs from Adult versus Prepubertal Sheep

**Figure 2:**
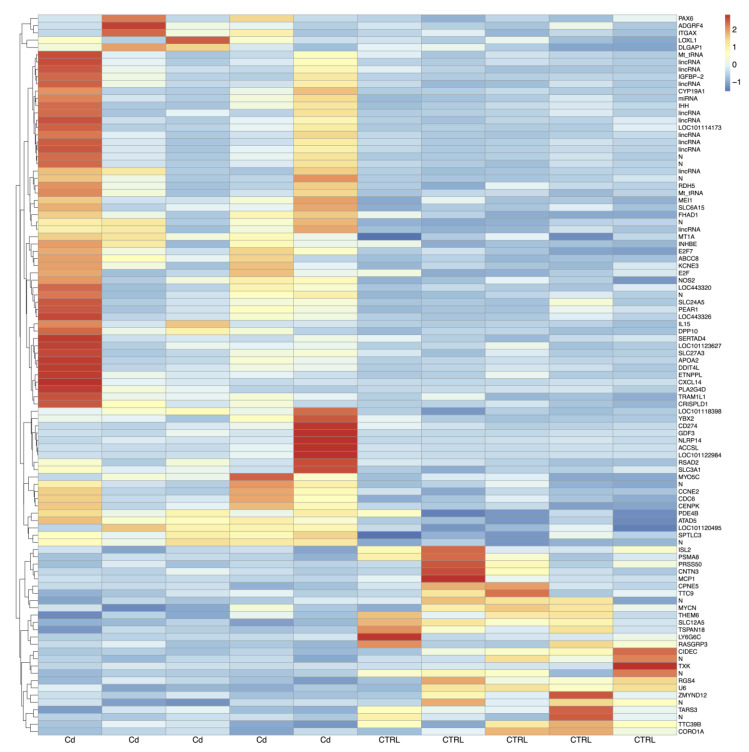
Heatmaps of DEGs in Cd-exposed versus control CCs from adult sheep. Blue to red colouration denotes low to high expression levels, respectively. Abbreviations: Cd, cadmium; CTRL, controls.

From CCs isolated from matured oocytes of adult sheep, on average, 23.5 million pairs of stranded reads per sample were obtained. About 83% of these reads were uniquely aligned to the reference genome (Ovis_aries.Oar_V3.1) and assigned to known mRNA transcripts. A total of 17,606 gene loci out of 20,028 Ensembl annotations were found expressed in CCs cells. By using unique and concordant RNA-seq reads, ninety-nine genes appeared differentially expressed (DEG) upon Cd exposure and are shown in the heatmap of Figure 2. Of these, 73 genes were upregulated (Table S1) whereas 26 genes were downregulated (Table S2) after in vitro exposure to Cd.

**Figure 3:**
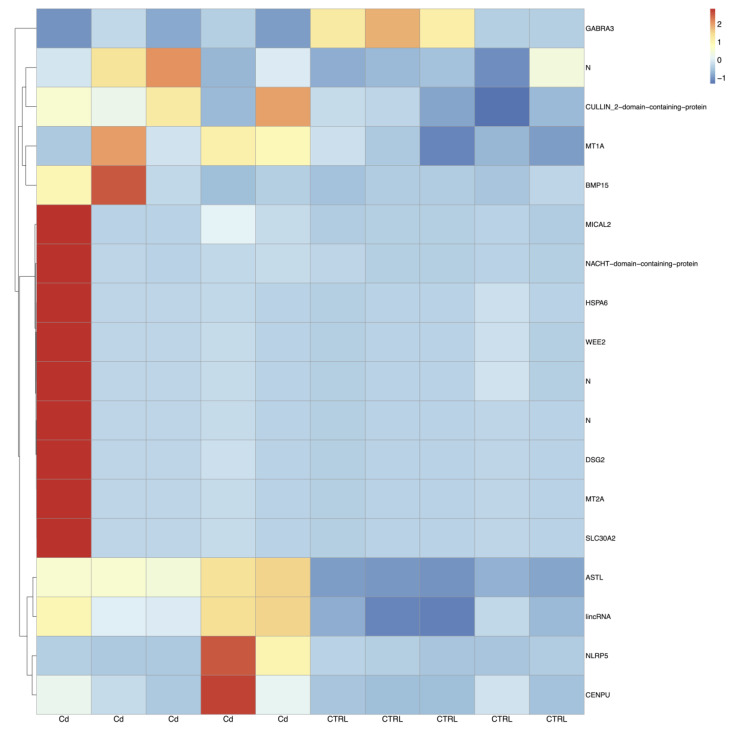
Heatmaps of DEGs in prepubertal CCs between the Cd-induced group and control group. Blue to red colouration denotes low to high expression levels, respectively. Abbreviations: Cd, cadmium; CTRL, controls.

RNA-seq of CCs isolated from matured oocytes of prepubertal lambs produced, on average, 64 million pairs of stranded reads per sample. After the removal of low-quality regions, about 80% of cleaned reads were uniquely aligned to the sheep reference genome and assigned to known gene loci. On the whole, 12,201 genes over the 20,028 known annotations were found expressed. Differential gene expression analysis revealed a lower number of DEGs (*n* = 18) upon Cd exposure in CCs of oocytes from prepubertal lambs compared to adults, as shown in the heatmap of Figure 3. Compared to controls, 17 genes appeared upregulated and only one was downregulated after Cd exposure (Table S3). To better characterize the functional role of DEG genes in both age groups, our attention was focused on protein-coding genes having an orthologue in humans through the GeneCards database.

Among adult upregulated genes, 46 were found in the GeneCards database. Of them, six DEGs met all three search criteria (IGFBP2, ITGAX, NOS2, PLA2G4D, SLC27A3 and YBX2) as they were found to be expressed in the human normal ovary, both at mRNA and protein level, and were found to be associated with PUBMED literature data on expression/function at ovarian level. As well, 17 DEGs (APOA2, CCNE2, CDC6, CENPK, CXCL14, CYP19A1, DDIT4L, GDF3, IHH, IL15, INHBE, LOXL1, MEI1, MT1A, MYO5C, NLRP14 and RSAD2) met two of the three criteria as they were found to be expressed in human normal ovary at mRNA level and were associated with protein data from GeneCards (Moped or ProteomicsDB) or with RNA/protein data from PUBMED. The remaining 23 DEGs (ABCC8, ACCSL, ADGRF4, ATAD5, CD274, CRISPLD1, DLGAP1, DPP10, EF21, E2F7, ETNPPL, FHAD1, KCNE3, PAX6, PDEA4B, PEAR1, SERTAD4, SLC3A1, SLC6A15, SLC24A5, SPTLC3, RDH5, TRAM1L1) displayed only mRNA expression in human normal ovary in the GeneCards database (Table S1).

Among adult downregulated genes, 21 were found in the GeneCards database. Of them, three DEGs (CIDEC, LY6G6C, and TSPAN18) met all three search criteria and 11 DEGs (CORO1A, MCP1, MYCN, PRSS50, PSMA8, RGS4, TARS3, THEM6, TTC39B, U6, and ZMYND12) responded to two of the three criteria. The remaining seven DEGs (CNTN3, CPNE5, ISL2, RASGRP3, SLC12A5, TTC9 and TXK) displayed only mRNA expression in human normal ovary in the GeneCards database (Table S2).

Among prepubertal DEGs, 14 genes were found in the GeneCards database and all of them were upregulated. Of them, three DEGs (CULLIN 2, DSG2 and SLC30A2) met all three search criteria (mRNA and protein expression in normal human ovary on GeneCards and PUBMED literature) and four DEGs (ASTL, BMP15, NLRP5, WEE2) were included in two of the three criteria, being expressed in human normal ovary at mRNA level and associated with protein data from GeneCards (Moped or ProteomicsDB) or with RNA/protein data from PUBMED literature. The remaining seven DEGs (CENPU, GABRA3, HSPA6, MICAL2, MT1A, MT2A, NACHT) displayed only mRNA expression in human normal ovary (Table S3).

Some adult upregulated genes known as being involved in various pathways of CC expansion and oocyte maturation, such as CYP19A1 (aromatase), IGFBP2, (insulin-like growth factor binding protein 2), NOS2 (nitric oxide inducible synthase) and IHH (Indian hedgehog signalling molecule) coding for proteins involved in the regulation of metabolic processes, cell signalling, growth and differentiation, and MT1A (metallothionein 1A), involved in ion transport, were chosen for subsequent enrichment and validation analysis.

In addition, some prepubertal overexpressed genes, known to be involved in various pathways of CC development and viability and CC-oocyte communications, such as SLC30A2 (Solute Carrier family 30 member 2) coding for a protein involved in the regulation of metal ions transport; HSPA6 (Heat Shock Protein family A member 6, Hsp70) having a role in chaperone-mediated protein folding; DSG2 (desmoglein 2) involved in cell-to-cell contact and BMP15 (Bone Morphogenetic Protein 15) involved in the regulation of cumulus-oocyte interaction crucial for fertilization, together with MT1A and MT2A (metallothionein 1A and 2A) involved in heavy metal detoxification, were used in subsequent enrichment and validation analysis.

### 3.4. Gene Networks Identifies Different Cd-Induced Pathways in Adult versus Prepubertal CCs

**Figure 4:**
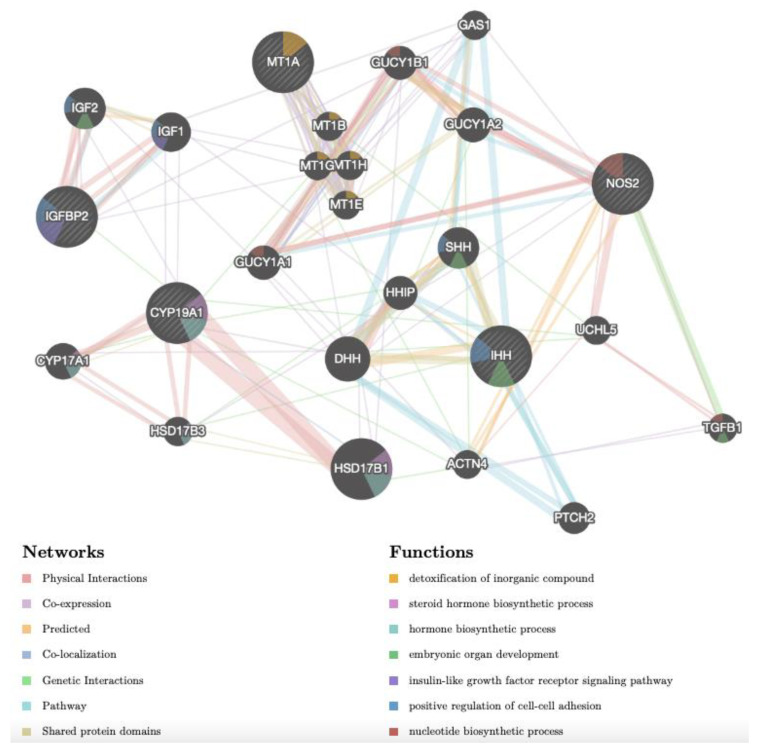
Gene network of DEGs (NOS2, IGFBP2, CYP19A1, IHH, MT1A), selected after transcriptomic analysis upon Cd treatment in CCs of adult sheep and analysed by GeneMANIA (http://www.genemania.org, accessed on 14 December 2022). The differently coloured the lines indicate the bioinformatics methods applied: co-expression, website prediction, pathway, physical interactions, and co-localization. The differently coloured network nodes indicate the biological functions of the set of enrichment genes.

Different gene networks were identified in CCs of adult and prepubertal lambs, indicating that different pathways were activated upon Cd exposure in the two age groups. The gene network of adult upregulated genes CYP19A1, IHH, IGFBP2, MT1A and NOS2 is shown in Figure 4. The network revealed complex interactions among the target genes and between them and other genes (Table S4). Considering the functions, some of these genes were reported to be associated with hormone and steroid hormone biosynthetic processes (CYP19A1, CYP17A1, HSD17B1 and HSD17B3), insulin-like growth factor receptor signalling pathway (IGF1 and IGFBP2), detoxification of inorganic compound (MT1A, MT1B, MT1E, MT1G, MT1H), cell–cell adhesion (IGF1, IGF2, IGFBP2, IHH and SHH) and embryo development (IGF2, IHH, TGFB1 and SHH). Considering the network, it can be seen that: (1) CYP19A1 has physical interactions with CYP17A1, HDS17B1 and HSD17B3 and it is co-expressed and/or has genetic interaction with several proteins of this network; (2) IGFBP2 has physical interactions and pathway interactions with IGF1 and IGF2; (3) NOS2 has various interactions, such as with TGFB1 (genetic interaction), GUCY1A2, GUCY1A1, GUCY1B1 and UCHL5 (physical interactions and pathways), ACTN4 (predicted); (4) IHH has different interactions, such as with PTCH, GAS1 and HHIP (pathways), DHH and SHH (predicted); (5) MT1A shows co-expression with four further metallothioneins.

**Figure 5:**
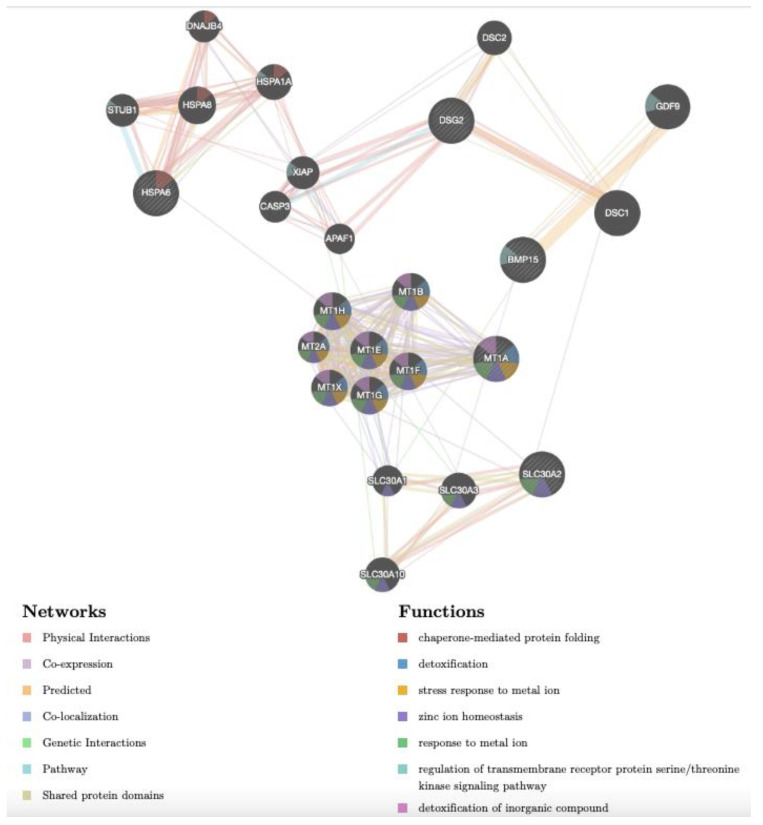
Gene network of DEGs (DSG2, MT1A, BMP15, SLC30A2, HSPA6), selected after transcriptomic analysis upon Cd treatment in CCs of prepubertal lambs and analysed by GeneMANIA (http://www.genemania.org, accessed on 14 December 2022). Differently coloured lines indicate the bioinformatics methods applied: co-expression, website prediction, pathway, physical interactions, and co-localization. The differently coloured network nodes indicate the biological functions of the set of enrichment genes.

The gene network of prepubertal upregulated genes BMP15, DSG2, HSPA6, MT1A and SLC30A2 is shown in Figure 5. As for adult samples, in prepubertal samples, the network revealed the same complex interactions among the target genes and between them and other genes (Table S4). Considering the functions, some of these genes were reported to be associated with chaperone-mediated protein folding (HSPA6, HSPA8, HPSA1A, STUB1 and DNAJB4), detoxification of inorganic compounds and stress response to metal ion (MT1A, MT2A, MT1B, MT1E, MT1F, MT1G, MT1H, MT1X), response to metal ion and zinc ion homeostasis (MT family, SLC30A1, SLC30A2, SLC30A3 and SLC30A10), regulation of transmembrane receptor protein serine/threonine kinase signalling pathway (GDF9, BMP15, HSPA1A and STUB1). Concerning the network, it can be seen that: (1) MT1A shows co-expression with seven further metallothioneins, (2) SLC30A2 shows various physical interactions with three other members of the SLC30 family; (3) DSG shows physical interactions with CASP3, XIAP, APAF1, DSC1 and DSC2; (4) BMP15 shows predicted interactions with GDF9; (5) HSPA6 shows physical interactions with other members of the same family (HSPA8 and HSPA1A) and with STUB1 and DNAJB4.

### 3.5. Cd-Induced CC Expression Pattern Is Consistent with the Sequencing Results

**Figure 6:**
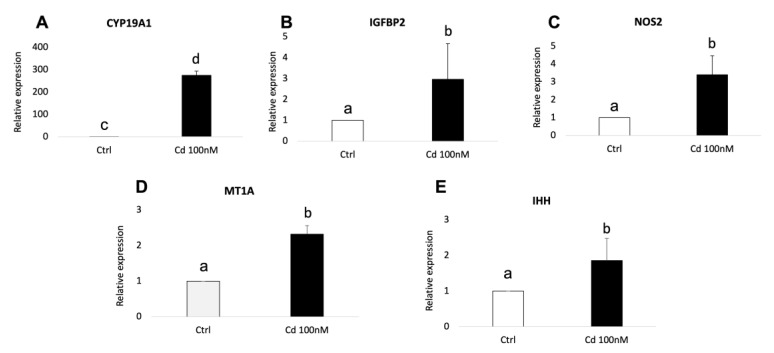
Relative expression of DEGs in CCs of adult sheep evaluated by Real-Time PCR. CYP19A1 (**A**), IGFBP2 (**B**), NOS2 (**C**), MT1A (**D**) and IHH (**E**) were upregulated upon CC exposure to 100 nM of CdCl_2_. Student’s *t*-test: a,b *p* < 0.05; c,d *p* < 0.01.

**Figure 7:**
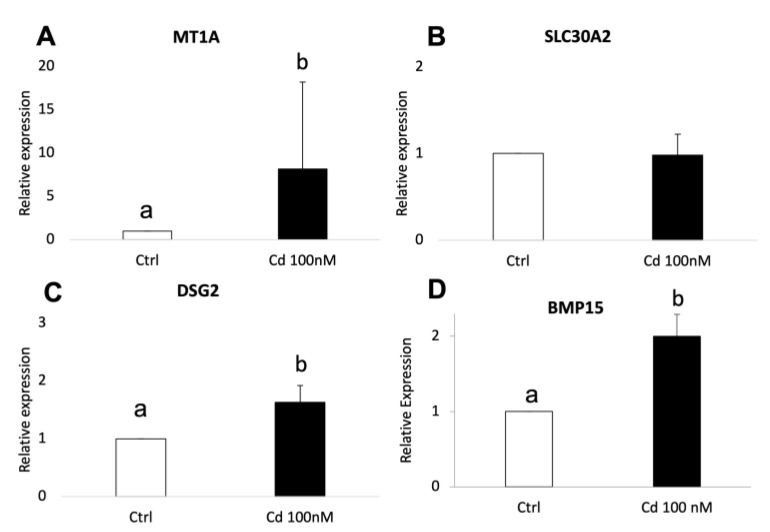
Relative expression of DEGs in CCs of prepubertal lambs evaluated by Real-Time PCR. MT1A (**A**), DSG2 (**C**) and BMP15 (**D**) were upregulated upon CC exposure to 100nM of CdCl_2_. No significative difference was found for SLC30A2 (**B**). Student’s *t*-test: a,b *p* < 0.05.

The validation of RNA-seq results was performed by quantitative RT-PCR using three independent biological replicates for each experimental condition (adult Cd-treated, adult controls, prepubertal Cd-treated, prepubertal controls). In adult samples, significantly increased expression of CYP19A1 (*p* < 0.01), NOS2 (*p* < 0.05), IGFBP2 (*p* < 0.05), MT1A (*p* < 0.05) and IHH (*p* < 0.05) was found in Cd-exposed CCs compared with controls (Figure 6, panels A–E). The expression pattern of all examined genes was in agreement with the DEGs profile. As well, in prepubertal samples, significantly increased expression of MT1A, DSG2 and BMP15 was found in Cd-exposed CCs compared with controls, in accordance with transcriptomic profile obtained after RNA-seq (*p* < 0.05; Figure 7, panels A–D). No significant differences were found for SLC30A2.

## 4. Discussion

This study analysed effects of in vitro exposure of COCs of the sheep model to environmental nanomolar Cd levels on transcriptomic profile of CCs in order to identify non-invasive biomarkers of Cd-induced oocyte dysfunction. To the best of our knowledge, this is the first study reporting the effects of this specific endocrine disruptor on CC transcriptome of a mammalian animal species. Previous studies analysed its effects on ovarian transcriptome in invertebrate species [45,46,47]. Methodological strengths of this reproductive toxicology study include: (1) the use of COCs of two donor age-groups, adult and prepubertal, recovered from two phases of female reproductive life in which oocytes are characterized by higher and lower developmental competence for adult and prepubertal respectively and, possibly, by different ability to respond to Cd-induced stress; (2) the exposure of COCs at environmental nanomolar Cd concentration, chosen within the range detected in ovarian tissues of prepubertal (0–2.7 ng/g ≅ 0–24.02 nM) and adult (7.89–24.39 ng/g ≅ 70.19–216.99 nM) sheep [16] and (3) the choice of exploring the transcriptomic dynamics in CCs which are considered as valuable non-invasive markers for MII oocyte quality [24,25,26,27,28,29,30,31,32,33,34,35,36].

Regarding IVM results, our data are in agreement and confirm those reported in our previous study in which 100 nM Cd did not affect oocyte maturation rate but decreased fertilization [16]. Similarly, in another study [22], exposure of bovine oocytes, during IVM, to 200 nM Cd did not affect oocyte nuclear maturation rate but reduced the cleavage and blastocyst rate compared with controls, indicating functional damage to the matured oocyte. As well, in porcine oocytes, exposure to 400 nM Cd did not affect oocyte maturation rate [23]. To the best of our knowledge, all other studies published to date that have found Cd deleterious effects of Cd on oocyte maturation have examined the effects of higher micromolar Cd concentrations [17,18,19,20,21,22,23,48]. In humans, in agreement with these studies, negative association of follicular fluid Cd nanomolar environmental levels with the probabilities of pregnancy and live birth was found [15].

Based on this large body of previous studies reporting that exposure to nanomolar Cd does not affect maturation, but rather fertilization, we decided to perform a transcriptomic analysis for exploring the molecular signature underlying Cd-induced impaired fertilization. At first glance it emerged that, from the quantitative point of view, in vitro exposure to environmental nanomolar Cd did not induce an excessively significant response in the number of DEGs involved. This is a positive finding considering that this environmental pollutant significantly affects animal and human fertility (See Introduction for References). Indeed, in comparison with recent, even non toxicological, transcriptomic studies on CCs by RNA-seq [27,49], the involvement of a hundred DEGs in adult and 18 DEGs in prepubertal CCs could be considered as indicative of a mild molecular response. However, the observed response should not be underestimated considering the variety and types of altered gene functions playing important roles in the development of oocyte ability to be fertilized. Some of these genes are confirmed by previous studies whereas others are found to be entirely new, as they were never previously associated with COC development and functions. Thus, they represent novel findings from this study that allow knowledge advancement and implementation of the available literature on molecular mechanisms underlying the acquisition of synchronized COC developmental competence.

Moreover, the different intensity of response between the two analysed age groups (higher number of DEGs in adult and lower number in prepubertal; see heatmaps and PCA analysis) led us to consider that these altered functions could be responsible for a full-blown condition in adults (Cd-induced infertility) whereas they could lead to a predisposition in prepubertal subjects (susceptibility to Cd-induced toxicity) in the used animal model. Furthermore, we can observe that basically the response has been mainly of upregulation both in adults and in prepubertal CCs. In our opinion, this could mean that the cells mainly tried to activate defence mechanisms (upregulated genes) and that a true inhibitory effect, at the tested Cd concentration, occurred on a lower number of genes (downregulated genes). Therefore, it could be hypothesized that exposure to this Cd concentration does not definitively damage the COC. Rather, its effects could be reversible, and detoxification strategies could be tested, as reported in a previous study from our group [48]. On the other hand, it cannot be excluded that some of upregulated genes could play inhibitory functions.

Among 46 adult upregulated genes found in the GeneCards database, 17 DEGs were previously associated with studies on cumulus/granulosa/oocyte functions reported in PUBMED. They include regulators of the cell cycle (CCNE2, CDC6); inflammation processes crucial for ovulation (CXCL14, IL15, ITGAX); steroid synthesis (CYP19A1); follicle/COC growth and differentiation (GDF3, IGFBP2, IHH, PLA2G4D); meiotic spindle organization (MEI1); periovulatory processes (MT1A, NLRP14, RSAD2); energetic homeostasis (SLC27A3); oxidative stress and apoptosis (NOS2); and stability and/or translation of germ cell mRNAs (YBX2). Given that some of these genes code for proteins that perform two or more of the aforementioned functions, the results of the present study confirm their expression and functional role in CCs of adult COCs. Moreover, the fact that these genes were overexpressed upon Cd exposure led us to hypothesize that they code for functions useful to prevent COC degeneration, thus preserving it for fertilization. Additional 6 DEGs were reported in GeneCards as expressed both at RNA and protein level in the ovary but, to our knowledge, no studies are available on PUBMED on their functional role in the COC. In light of their ovarian expression, their possible role and involvement in COC preparation to the fertilization process can be hypothesized as they are reported to code for several functions, such as: cholesterol transport (APOA2), component of the centromere (CENPK), signal transduction regulation (DDIT4L), cell proliferation, apoptosis, immune response and hormone secretion regulation (INHBE), extracellular matrix remodelling (LOXL1), actin filament organization and vesicle transport (MYO5C). The remaining 23 upregulated genes have not been annotated previously in studies on oocyte maturation and fertilization (Table S1 [50,51,52,53,54,55,56,57,58,59,60,61,62,63,64,65,66,67,68,69,70,71,72,73,74,75,76,77,78,79,80,81,82,83,84,85,86,87,88,89,90,91,92]).

Among 21 adult downregulated genes found in the GeneCards database, seven DEGs were associated with previous studies on cumulus/granulosa/oocyte functions reported in PUBMED. They include regulators of the cell cycle (MYCN), apoptosis (CIDEC), ovarian inflammatory processes (LY6G6C, MCP1), G protein signalling (RGS4), sperm-egg fusion (TSPAN18) and post-transcriptional modifications in oocyte target genes (U6). Additionally, six DEGs were reported in GeneCards as expressed both at RNA and protein level in the ovary but, to our knowledge, not in previous PUBMED studies. Based on their ovarian expression, it could be hypothesized that they play a role in COC functions as they are reported to code for: cell cycle progression, signal transduction and apoptosis (CORO1A, PSMA8), proteolysis (PRSS50), thioesterase activity (THEM6), cholesterol homeostasis (TTC39B), metal ion binding activity (ZMYND12). The remaining 8 DEGs (CNTN3, CPNE5, ISL2, RASGRP3, SLC12A5, TARS3, TTC9, TXK), displaying only mRNA expression in human normal ovary, have never been annotated in previous studies on COC competence (Table S2 [93,94,95,96,97,98,99,100,101,102,103,104,105,106,107,108]).

Among 14 prepubertal upregulated genes found in the GeneCards database, three DEGs were associated with previous studies on cumulus/granulosa/oocyte functions reported in PUBMED. They include genes coding for proteins involved in the regulation of: protein ubiquitination essential for germline development (CULLIN 2), follicle development through the regulation of cell-cell junctions (DSG2 Desmoglein 2), zinc homeostasis in the oocyte (SLC30A2, Solute Carrier Family 30 member 2), sperm-zona binding and prevention of polyspermy (ASTL, Astacin Like Metalloendopeptidase), cumulus-oocyte communications (BMP15, Bone morphogenetic protein 15 and NLRP5, known as MATER), oocyte meiotic arrest (WEE2, Oocyte meiosis inhibiting kinase). The remaining seven DEGs (CENPU, GABRA3, HSPA6, MICAL2, MT1A, MT2A, NACHT) displayed only mRNA expression in human normal ovary and have never been annotated in previous studies on COC competence (Table S3 [28,79,109,110,111,112,113,114,115,116,117,118,119,120,121,122,123,124,125,126,127,128,129,130,131,132,133,134,135,136,137]).

Overall, the 23 upregulated and eight downregulated genes from adult CCs and the seven upregulated genes from prepubertal CCs, not yet annotated in previous study on COC functions, represent a novel group of genes captured by this study design implicated in its developmental competence. These DEGs should be further explored as they have never been previously reported in such processes.

DEGs used for enrichment and or validation analysis were chosen among upregulated ones and having known role in ovarian function or known response to Cd. They have been analysed to identify their possible role in the modulation of the COC response to Cd.

Concerning DEGs overexpressed in adult CCs, in the present study, significant CYP19A1 upregulation was found after Cd exposure, with an increase of about 250 times greater in Cd-exposed samples than in controls. This finding could mean that, in CCs, Cd may have acted as an endocrine disruptor, as the upregulation of CYP19A1, coding for aromatase, the most important enzyme involved in oestrogen biosynthesis, may have led to excess oestrogen levels, which is known to be related to deleterious effects on follicular development, oocyte and embryo quality [138]. This finding is in agreement with that of a previous study in mice, in which high levels of steroid hormones were found upon Cd exposure contributing to female sexual dysfunction with premature puberty onset [139]. However, CYP19A1 downregulation was found in response to Cd in other studies. In carp, it was showed that Cd affected steroidogenesis in a dose and time-dependent manner by reducing aromatase activity and expression [140]. Similarly, in another study, downregulation of aromatase expression in zebrafish female ovaries was found upon Cd exposure [46]. This apparent discrepancy on effects of Cd on aromatase expression could be linked to the different organism/cell regulation systems or Cd tested concentration.

According with deep sequencing results, Cd exposure increased the gene expression of IGFBP2 in CCs of adult sheep. Considering its function in ovarian follicles [141], the observed upregulation of IGFBP2 could lead to negative modulation of steroidogenesis in GCs and follicular atresia. To the best of our knowledge, this is the first study reporting the modulation of IGFBP2 expression following Cd exposure; therefore, our data can be discussed in the light of studies conducted in other cell systems or under different experimental conditions. IGFBP2 has been shown to block FSH-dependent E2 production in the ovarian follicle [64]. In our study, it cannot be excluded that the IGFBP2 overexpression could be a feedback mechanism induced by the excessive expression of CYP19A1 and the subsequent synthesis of oestrogens after Cd exposure.

The NOS2 gene, encoding for inducible nitric oxide synthase, was also upregulated by exposure to Cd in our study. It could be assumed that Cd damage on CCs is associated to inflammatory and oxidative state, as this gene is usually activated by inflammatory molecules or toxic elements. To the best of our knowledge, there are no studies that have analysed the effects of Cd on the expression of NOS2 in CCs; therefore, our data can be discussed in the light of studies conducted in other cell systems or under different experimental conditions. It has been demonstrated that Cd upregulated NOS2, resulting in increased nitric oxide (NO) production implicated in Cd-mediated cytotoxicity in mouse macrophages [142] and arteria [143], and in rat liver [144] and testis [145]. In human CCs, NOS mRNA expression has been negatively correlated with oocyte receptibility to fertilisation, as increased NOS2 expression was found in non-fertilized oocyte after ICSI compared to fertilized oocyte, so it could be considered a negative marker of oocyte competence [83]. On steroidogenesis, NO synthesis can directly inhibit aromatase both in human GCs and luteal cells [146] and this inhibitory effect on steroidogenesis can be considered a feedback mechanism induced by the excessive expression of CYP19A1 and the subsequent synthesis of oestrogen after Cd exposure.

The IHH gene, encoding for the Indian Hedgehog Signalling Molecule, was upregulated by exposure to Cd in our study. This signalling pathway has been shown to be involved in the regulation of several cellular activities, such as protein metabolic processes in bovine CCs [69], apoptosis in swine GCs [70] and GC differentiation in mice [73]. In our study, overexpression upon Cd exposure might indicate a response with a double meaning. On one side, IHH may have triggered an apoptotic process, since in a previous study, IHH was upregulated during the time of in vitro culture of the GCs, demonstrating an association with processes of aging and programmed GC death [70]. On the other side, Cd may have acted as an endocrine disruptor. In fact, in a previous study on women with polycystic ovary syndrome (PCOS), one of the most common endocrine disorders in women, IHH was abnormally highly expressed in the PCOS tissue [72]. To the best of our knowledge, the only study investigating IHH levels in relation to Cd effects was performed on zebrafish swim bladder [147]. Further studies are needed to clarify Cd effects on Hedgehog signalling pathway in CCs.

Among upregulated genes upon Cd exposure, there was MT1A involved in the regulation of metal ions transport. This finding is in agreement with previous studies in non-vertebrate organisms in which altered expression of the same gene or genes belonging to the same family were found, using transcriptomic approaches, in ovarian tissues [47]. Indeed, increased expression of MT1A was found which could be considered as a defensive cellular response activated by CCs after Cd exposure. In fact, due to the high affinity with zinc ions, MT1 plays an important role in the detoxification from heavy metals and free radicals produced during oxidative stress conditions [148]. In our experimental conditions, MT1A gene could have been overexpressed to provide increased amount of this protein to sequester excessive Cd for detoxification. Our results are in agreement with those obtained in previous studies in the mouse model in which increased expression of MT1 was observed in response to Cd absorption in liver [149], kidney, spleen, lung and heart, and this mechanism protected these organs from Cd toxicity [150]. In another study, uterine cells displayed increased expression of MT1A gene after Cd exposure, and this overexpression has been related to steroid estrogenic-dependent regulation [151]. Considering our findings, we could hypothesize that the promoted expression of MT1A in Cd-exposed CCs is a protective response of CCs against Cd-toxicity. Indeed, MT1A is a zinc-binding protein. When Cd is present, it binds to MT1A thus competing with zinc ions (ionic mimicry, namely the ability of a cationic form of a toxic metal to mimic an essential element or cationic species of an element at the site of a transporter of that element; [152] and removing Cd from the cell. Interestingly, this DEG was upregulated in adult and in prepubertal CCs. This finding led us to identify it as the most reliable biomarker of Cd-induced CC toxicity. Furthermore, MT2A, another member of metallothionein family, was found to be overexpressed. This gene also codes for a protein involved in homeostatic control and detoxification of heavy metals. Moreover, thanks to enrichment analysis, in adult and in prepubertal CCs, the involvement of many other members of MT family has been evidenced upon Cd exposure.

Continuing among overexpressed genes in prepubertal CCs, we focused our attention on HSPA6, BMP15, DSG2 and SLC30A2. Although HSPA6 has been observed as deregulated in several studies upon Cd exposure [153,154,155,156], another study reported its overexpression in Cd-exposed human iPSC-derived renal cells, suggesting that other stressors (e.g., Cd) in addition to the heat stress, could regulate HSPA6 expression levels [157]. Similarly, HSPA6 expression was significantly upregulated in response to Cd exposure in human trophoblast cells [158], as a clear indication of activation of a defence system in response to a stressful factor.

Different studies have demonstrated that BMP15 is essential for normal follicular development [111,112,113] and for the acquisition of oocyte competence [159], through an efficient communication between the oocyte and its surrounding somatic cells [28,114]. Recently, BMP15 was also found to be overexpressed in the CCs of PCOS patients following drug treatments [160]. In our study, we identified a never shown before expression in CCs upon Cd exposure. The enrichment analysis identified co-expression with SLC30A3 and genetic interaction with SLC30A1; therefore, we could hypothesize that BMP15 overexpression in CCs was a stress-mediated response to the metal ion Cd, in addition to the other well-known detoxification systems mentioned already. To the best of our knowledge, to date, no studies have investigated the expression of this gene in relation to Cd effects, even in other cell systems, so further studies are needed to clarify these events.

DSG2 code for a desmoglein, a component of desmosomes, cell-cell junctions which have been reported as involved in apoptosis, upregulated genes in GCs from atretic follicles [116]. To the best of our knowledge, no previous studies have reported to date on effects of Cd on the expression of this gene, but its role in Cd-mediated CC toxicity can be hypothesized as it is well known that Cd affects intercellular cumulus communications and regular expansion.

According to deep sequencing results, Cd exposure increased the gene expression of SLC30A2 in CCs of prepubertal lambs. The Solute Carrier Family 30 Member 2 (SLC30A2) is a Zn transporter involved in Zn homeostasis within the cells [161]. Several studies have suggested that the toxic effects of Cd may be mediated by altered metabolism of essential elements, including Zn [162]. Zn homeostasis plays a pivotal role in the functioning of female germ cells. In murine COCs, there is a strict control of Zn levels within the oocyte supported by CCs [131], which actively regulate the uptake of Zn into the oocyte during maturation, which is necessary for the completion of meiosis and for MII arrest. In a previous study, Cd-exposure of rat mother caused depletion of Zn and upregulation of SLC30A2 gene expression in gastrointestinal tract and plasma of suckling puppets [162]. The validation procedure did not identify differences in the expression levels of SLC30A in CCs treated with Cd compared to controls. This result could be related to the use of independent CC samples for validation and transcriptomic analysis, which, although conducted under the same culture conditions, were derived from ovaries of different animals.

Interestingly, for all these genes, the enrichment analysis, in adult CCs, revealed interactions among all aforementioned genes involved in COC growth/differentiation, functional and detoxification pathways in response to Cd. Instead, in prepubertal CCs, independent clusters of genes, involved essentially in detoxification functions (MT family and SLC30) were identified. This difference could be explained considering that in prepubertal CCs, some functions could be not yet expressed or active (Table S4).

As a final consideration, downregulation was a lower intense response in this study, as it accounted for 23% of dysregulated genes. However, no less importance should be attributed to the findings of significantly downregulated genes upon Cd exposure among which, in particular, the presence of MYNC stands out as associated with oocyte maturation pathways [103], along with RGS4, which is downregulated in women with diminished ovarian reserves [105], and TSPAN18, which is involved in facilitating sperm inner acrosomal membrane apposition with the oolemma [107] (See Table S2 for further details).

## 5. Conclusions

In conclusion, our findings identified genes over- and under-expressed upon in vitro exposure to nanomolar Cd in CCs of adult and prepubertal oocytes in sheep. For genes previously associated with COC molecular developmental competence, the results of this study confirm and implement the knowledge on their involvement in these processes. Moreover, the novel identified genes can serve as a springboard to future studies. We plan to perform parallel studies aimed to identify, in humans and in the sheep model, shared biomarkers of Cd-induced infertility, to correlate them with clinical outcomes and develop appropriate detoxification strategies.

## Figures and Tables

**Table 1:** Primer pair sequences used for Real-Time PCR.

Gene Symbol	Full Name	Accession Number	Primer (5′→3′)	Ta (°C)	Size bps
ACTB	Actin beta	NM_001009784	CCCTGGAGAAGAGCTACGAG TAGTTTCGTGAATGCCGCAG	59	129
MT-1A	Metallothionein-1A	XM_004014996	CTTGCCACTTGTTCTGGACC AGCTCTTCTTGCAGGAGGG	59	139
DSG2	Desmoglein 2	XM_027960751.2	CCGCCTTTTGGTGTGTTTGT AAGCGTAGCCAGTTAGCAGA	60	106
SLC30A2	Solute carrier family (zinc transporter), member 2	XM_012151636.2	TCATCTGTGGGCTGAGAACG TCCACAACCACCATGTGCTC	59	90
BMP15	BMP15 bone morphogenetic protein 15	NM_001114767.2	TGGTCCTCCTGAGCATCCTT CTCTGAGAGGCCTTGCTACA	60	312
CYP19A1	Aromatase	NM_001123000.1	CTCTCCTTCTCAAACCAGACATCTT ATGGCATCTTTCAAGTCCTTGACA	59	88
NOS2	Nitric Oxide Synthase 2	XM_012185382.3	AGAGACGGGGAGATCGGAAA TGGGGATCTCAATGTGGTGC	59	452
IGFBP2	Insulin-like Growth Factor Binding Protein 2	NM_001009436.1	GTGGCAAACATCACCTTGGC CCAGTGTTGGGGTTCACACA	60	259
IHH	Indian hedgehog signaling molecule	XM_027965204.1	CACGGCCAACAATCACACTG CCCATGCCAAGCTGTGAAAC	60	284

**Table 2:** Effects of in vitro exposure to nanomolar Cd during IVM on meiotic progression and maturation of oocytes from adult sheep and prepubertal lambs.

Age Group	Cd Concentration (nM)	N° Evaluated Oocytes	Nuclear Chromatin Configurations (n°, %)			
			GV	MI a TI	MII + PB	Abnormal
Adult	0	197	2 (1.0)	6 (3.0)	187 (95.0)	2 (1.0)
	100	204	6 (3.0)	8 (3.9)	181 (88.7)	9 (4.4)
Prepubertal	0	180	4 (2.2)	10 (5.6)	161 (89.4)	5 (2.8)
	100	161	6 (3.7)	5 (3.1)	129 (87.6)	9 (5.6)

Table legend: GV = Germinal Vesicle; MI = Metaphase I; TI = Telophase I; MII = Metaphase II; PB = Polar Body. Chi-square test: Not significant. Seven and eight replicates were performed for adult and prepubertal groups, respectively. A replicate included CCs from 20–30 COCs, each of them individually cultured in 10 μL/microdrop placed in a 60 mm petri dish and selected after IVM for belonging to a MII oocyte.

## Data Availability

The data presented in this study are available on request from the corresponding author.

## Supplementary Materials

The following are available online at https://www.mdpi.com/article/10.3390/biology12020249/s1, Table S1: Upregulated genes in CCs of adult sheep exposed in vitro to nanomolar cadmium, Table S2: Downregulated genes in CCs isolated from adult sheep and exposed in vitro to nanomolar cadmium, Table S3: Upregulated genes in CCs isolated from prepubertal lambs and exposed in vitro to nanomolar cadmium, Table S4: Detailed results of the gene network analysis.

## References

[1] Canipari R., De Santis L., Cecconi S. (2020). Female Fertility and Environmental Pollution. Int. J. Environ. Res. Public Health.

[2] Wrzecińska M., Kowalczyk A., Cwynar P., Czerniawska-Piątkowska E. (2021). Disorders of the Reproductive Health of Cattle as a Response to Exposure to Toxic Metals. Biology.

[3] Rzymski P., Tomczyk K., Rzymski P., Poniedziałek B., Opala T., Wilczak M. (2015). Impact of Heavy Metals on the Female Reproductive System. Ann. Agric. Environ. Med..

[4] Thompson J., Bannigan J. (2008). Cadmium: Toxic Effects on the Reproductive System and the Embryo. Reprod. Toxicol..

[5] Flora S.J.S., Agrawal S. (2017). Arsenic, Cadmium, and Lead. Reproductive and Developmental Toxicology.

[6] Everson T.M., Punshon T., Jackson B.P., Hao K., Lambertini L., Chen J., Karagas M.R., Marsit C.J. (2018). Cadmium-Associated Differential Methylation throughout the Placental Genome: Epigenome-Wide Association Study of Two U.S. Birth Cohorts. Environ. Health Perspect..

[7] Zhu J.-Q., Liu Y., Zhang J.-H., Liu Y.-F., Cao J.-Q., Huang Z.-T., Yuan Y., Bian J.-C., Liu Z.-P. (2018). Cadmium Exposure of Female Mice Impairs the Meiotic Maturation of Oocytes and Subsequent Embryonic Development. Toxicol. Sci..

[8] Cheng Y., Zhang J., Wu T., Jiang X., Jia H., Qing S., An Q., Zhang Y., Su J. (2019). Reproductive Toxicity of Acute Cd Exposure in Mouse: Resulting in Oocyte Defects and Decreased Female Fertility. Toxicol. Appl. Pharmacol..

[9] Dong F., Li J., Lei W.-L., Wang F., Wang Y., Ouyang Y.-C., Hou Y., Wang Z.-B., Schatten H., Sun Q.-Y. (2020). Chronic Cadmium Exposure Causes Oocyte Meiotic Arrest by Disrupting Spindle Assembly Checkpoint and Maturation Promoting Factor. Reprod. Toxicol..

[10] Ruslee S.S., Zaid S.S.M., Bakrin I.H., Goh Y.M., Mustapha N.M. (2020). Protective Effect of Tualang Honey against Cadmium-Induced Morphological Abnormalities and Oxidative Stress in the Ovary of Rats. BMC Complement. Med. Ther..

[11] Tian J., Hu J., Liu D., Yin J., Chen M., Zhou L., Yin H. (2021). Cadmium Chloride-Induced Transgenerational Neurotoxicity in Zebrafish Development. Environ. Toxicol. Pharmacol..

[12] Zenzes M.T., Krishnan S., Krishnan B., Zhang H., Casper R.F. (1995). Cadmium Accumulation in Follicular Fluid of Women in in Vitro Fertilization-Embryo Transfer Is Higher in Smokers. Fertil. Steril..

[13] Piasek M., Blanuša M., Kostial K., Laskey J.W. (2001). Placental Cadmium and Progesterone Concentrations in Cigarette Smokers. Reprod. Toxicol..

[14] Wdowiak A., Wdowiak E., Bojar I. (2018). Evaluation of Trace Metals in Follicular Fluid in ICSI-Treated Patients. Ann. Agric. Environ. Med..

[15] Wu S., Wang M., Deng Y., Qiu J., Zhang X., Tan J. (2020). Associations of Toxic and Essential Trace Elements in Serum, Follicular Fluid, and Seminal Plasma with In Vitro Fertilization Outcomes. Ecotoxicol. Environ. Saf..

[16] Martino N.A., Marzano G., Mangiacotti M., Miedico O., Sardanelli A.M., Gnoni A., Lacalandra G.M., Chiaravalle A.E., Ciani E., Bogliolo L. (2017). Exposure to Cadmium during in Vitro Maturation at Environmental Nanomolar Levels Impairs Oocyte Fertilization through Oxidative Damage: A Large Animal Model Study. Reprod. Toxicol..

[17] Leoni G., Bogliolo L., Deiana G., Berlinguer F., Rosati I., Pintus P.P., Ledda S., Naitana S. (2002). Influence of Cadmium Exposure on in Vitro Ovine Gamete Dysfunction. Reprod. Toxicol..

[18] Mlynarcíková A., Scsuková S., Vrsanská S., Nagyová E., Ficková M., Kolena J. (2004). Inhibitory Effect of Cadmium and Tobacco Alkaloids on Expansion of Porcine Oocyte-Cumulus Complexes. Cent. Eur. J. Public Health.

[19] Lazzari G., Tessaro I., Crotti G., Galli C., Hoffmann S., Bremer S., Pellizzer C. (2008). Development of an in Vitro Test Battery for Assessing Chemical Effects on Bovine Germ Cells under the ReProTect Umbrella. Toxicol. Appl. Pharmacol..

[20] Nandi S., Gupta P.S.P., Selvaraju S., Roy S.C., Ravindra J.P. (2010). Effects of Exposure to Heavy Metals on Viability, Maturation, Fertilization, and Embryonic Development of Buffalo (*Bubalus bubalis*) Oocytes In Vitro. Arch. Environ. Contam. Toxicol..

[21] Tessaro I., Modina S.C., Crotti G., Franciosi F., Colleoni S., Lodde V., Galli C., Lazzari G., Luciano A.M. (2015). Transferability and Inter-Laboratory Variability Assessment of the in Vitro Bovine Oocyte Fertilization Test. Reprod. Toxicol..

[22] Akar Y., Ahmad N., Khalıd M. (2018). The Effect of Cadmium on the Bovine in Vitro Oocyte Maturation and Early Embryo Development. Int. J. Vet. Sci. Med..

[23] Zhou C., Zhang X., Chen Y., Liu X., Sun Y., Xiong B. (2019). Glutathione Alleviates the Cadmium Exposure-Caused Porcine Oocyte Meiotic Defects via Eliminating the Excessive ROS. Environ. Pollut..

[24] Gilchrist R.B., Ritter L.J., Armstrong D.T. (2004). Oocyte–Somatic Cell Interactions during Follicle Development in Mammals. Anim. Reprod. Sci..

[25] Matzuk M.M., Burns K.H., Viveiros M.M., Eppig J.J. (2002). Intercellular Communication in the Mammalian Ovary: Oocytes Carry the Conversation. Science.

[26] Bunel A., Jorssen E.P., Merckx E., Leroy J.L., Bols P.E., Sirard M.A. (2015). Individual Bovine in Vitro Embryo Production and Cumulus Cell Transcriptomic Analysis to Distinguish Cumulus-Oocyte Complexes with High or Low Developmental Potential. Theriogenology.

[27] Wyse B.A., Fuchs Weizman N., Kadish S., Balakier H., Sangaralingam M., Librach C.L. (2020). Transcriptomics of Cumulus Cells—A Window into Oocyte Maturation in Humans. J. Ovarian Res..

[28] Eppig J. (2001). Oocyte Control of Ovarian Follicular Development and Function in Mammals. Reproduction.

[29] Su Y.-Q., Sugiura K., Woo Y., Wigglesworth K., Kamdar S., Affourtit J., Eppig J.J. (2007). Selective Degradation of Transcripts during Meiotic Maturation of Mouse Oocytes. Dev. Biol..

[30] Eichenlaub-Ritter U., Vogt E., Yin H., Gosden R. (2004). Spindles, Mitochondria and Redox Potential in Ageing Oocytes. Reprod. Biomed. Online.

[31] Eppig J.J. (2018). Reproduction: Oocytes Call, Granulosa Cells Connect. Curr. Biol..

[32] Bachvarova R. (1985). Gene Expression During Oogenesis and Oocyte Development in Mammals. Oogenesis.

[33] Braude P., Bolton V., Moore S. (1988). Human Gene Expression First Occurs between the Four- and Eight-Cell Stages of Preimplantation Development. Nature.

[34] De La Fuente R., Viveiros M.M., Burns K.H., Adashi E.Y., Matzuk M.M., Eppig J.J. (2004). Major Chromatin Remodeling in the Germinal Vesicle (GV) of Mammalian Oocytes Is Dispensable for Global Transcriptional Silencing but Required for Centromeric Heterochromatin Function. Dev. Biol..

[35] Macaulay A.D., Gilbert I., Caballero J., Barreto R., Fournier E., Tossou P., Sirard M.-A., Clarke H.J., Khandjian É.W., Richard F.J. (2014). The Gametic Synapse: RNA Transfer to the Bovine Oocyte. Biol. Reprod..

[36] Macaulay A.D., Gilbert I., Scantland S., Fournier E., Ashkar F., Bastien A., Saadi H.A.S., Gagné D., Sirard M.-A., Khandjian É.W. (2016). Cumulus Cell Transcripts Transit to the Bovine Oocyte in Preparation for Maturation1. Biol. Reprod..

[37] Chronowska E. (2014). High-Throughput Analysis of Ovarian Granulosa Cell Transcriptome. Biomed. Res. Int..

[38] Hallberg I., Persson S., Olovsson M., Sirard M.-A., Damdimopoulou P., Rüegg J., Sjunnesson Y.C.B. (2021). Perfluorooctane Sulfonate (PFOS) Exposure of Bovine Oocytes Affects Early Embryonic Development at Human-Relevant Levels in an in Vitro Model. Toxicology.

[39] Martino N.A., Lacalandra G.M., Filioli Uranio M., Ambruosi B., Caira M., Silvestre F., Pizzi F., Desantis S., Accogli G., Dell’Aquila M.E. (2012). Oocyte Mitochondrial Bioenergy Potential and Oxidative Stress: Within-/between-Subject, inVivo versus in Vitro Maturation, and Age-Related Variations in a Sheep Model. Fertil. Steril..

[40] Mastrorocco A., Martino N.A., Marzano G., Lacalandra G.M., Ciani E., Roelen B.A.J., Dell’Aquila M.E., Minervini F. (2019). The Mycotoxin Beauvericin Induces Oocyte Mitochondrial Dysfunction and Affects Embryo Development in the Juvenile Sheep. Mol. Reprod. Dev..

[41] Dell’Aquila M.E., Asif S., Temerario L., Mastrorocco A., Marzano G., Martino N.A., Lacalandra G.M., Roelen B.A., Carluccio A., Robbe D. (2021). Ochratoxin A Affects Oocyte Maturation and Subsequent Embryo Developmental Dynamics in the Juvenile Sheep Model. Mycotoxin Res..

[42] Dobin A., Davis C.A., Schlesinger F., Drenkow J., Zaleski C., Jha S., Batut P., Chaisson M., Gingeras T.R. (2013). STAR: Ultrafast Universal RNA-Seq Aligner. Bioinformatics.

[43] Love M.I., Huber W., Anders S. (2014). Moderated Estimation of Fold Change and Dispersion for RNA-Seq Data with DESeq2. Genome Biol..

[44] Franz M., Rodriguez H., Lopes C., Zuberi K., Montojo J., Bader G.D., Morris Q. (2018). GeneMANIA Update 2018. Nucleic Acids Res..

[45] Su H., Yang Y., Zou J., Cheng Y., Yang Y., Wu J., Pollak P., Yang Y. (2020). Transcriptome Analysis of the Ovary of Beet Armyworm Spodoptera Exigua under Different Exposures of Cadmium Stress. Chemosphere.

[46] Liu P., Zhao Y., Wang S., Xing H., Dong W.-F. (2021). Effect of Combined Exposure to Silica Nanoparticles and Cadmium Chloride on Female Zebrafish Ovaries. Environ. Toxicol. Pharmacol..

[47] Wang J., Peng X., Yang H., Lv B., Wang Z., Song Q. (2020). Mul-Tiomics Analysis of Cadmium Stress on the Ovarian Function of the Wolf Spider Pardosa Pseudoannulata. Chemosphere.

[48] Piras A.R., Ariu F., Maltana A., Leoni G.G., Martino N.A., Mastrorocco A., Dell’Aquila M.E., Bogliolo L. (2022). Protective Effect of Resveratrol against Cadmium-Induced Toxicity on Ovine Oocyte in Vitro Maturation and Fertilization. J. Anim. Sci. Biotechnol..

[49] Liu Q., Zhang J., Wen H., Feng Y., Zhang X., Xiang H., Cao Y., Tong X., Ji Y., Xue Z. (2018). Analyzing the Transcriptome Profile of Human Cumulus Cells Related to Embryo Quality via RNA Sequencing. Biomed. Res. Int..

[50] Agca C., Yakan A., Agca Y. (2013). Estrus Synchronization and Ovarian Hyper-Stimulation Treatments Have Negligible Effects on Cumulus Oocyte Complex Gene Expression Whereas Induction of Ovulation Causes Major Expression Changes. Mol. Reprod. Dev..

[51] Yi Z., Meng T., Ma X., Li J., Zhang C., Ouyang Y., Schatten H., Qiao J., Sun Q., Qian W. (2020). CDC6 Regulates Both G2/M Transition and Metaphase-to-anaphase Transition during the First Meiosis of Mouse Oocytes. J. Cell. Physiol..

[52] Anger M., Stein P., Schultz R.M. (2005). CDC6 Requirement for Spindle Formation During Maturation of Mouse Oocytes1. Biol. Reprod..

[53] Okada M., Cheeseman I.M., Hori T., Okawa K., McLeod I.X., Yates J.R., Desai A., Fukagawa T. (2006). The CENP-H–I Complex Is Required for the Efficient Incorporation of Newly Synthesized CENP-A into Centromeres. Nat. Cell. Biol..

[54] Qi J., Li J., Wang Y., Wang W., Zhu Q., He Y., Lu Y., Wu H., Li X., Zhu Z. (2021). Novel Role of CXCL14 in Modulating STAR Expression in Luteinized Granulosa Cells: Implication for Progesterone Synthesis in PCOS Patients. Transl. Res..

[55] Bobe J., Montfort J., Nguyen T., Fostier A. (2006). Identification of New Participants in the Rainbow Trout (Oncorhynchus Mykiss) Oocyte Maturation and Ovulation Processes Using CDNA Microarrays. Reprod. Biol. Endocrinol..

[56] Costermans N.G.J., Soede N.M., Van Tricht F., Blokland M., Kemp B., Keijer J., Teerds K.J. (2020). Follicular Fluid Steroid Profile in Sows: Relationship to Follicle Size and Oocyte Quality†. Biol. Reprod..

[57] Liu T., Huang Y., Lin H. (2021). Estrogen Disorders: Interpreting the Abnormal Regulation of Aromatase in Granulosa Cells (Review). Int. J. Mol. Med..

[58] Motahari Rad H., Mowla S.J., Ramazanali F., Rezazadeh Valojerdi M. (2022). Characterization of Altered MicroRNAs Related to Different Phenotypes of Polycystic Ovarian Syndrome (PCOS) in Serum, Follicular Fluid, and Cumulus Cells. Taiwan J. Obstet. Gynecol..

[59] Heidarzadehpilehrood R., Pirhoushiaran M., Abdollahzadeh R., Binti Osman M., Sakinah M., Nordin N., Abdul Hamid H. (2022). A Review on CYP11A1, CYP17A1, and CYP19A1 Polymorphism Studies: Candidate Susceptibility Genes for Polycystic Ovary Syndrome (PCOS) and Infertility. Genes.

[60] Mukhopadhyay R., Prabhu N.B., Kabekkodu S.P., Rai P.S. (2022). Review on Bisphenol A and the Risk of Polycystic Ovarian Syndrome: An Insight from Endocrine and Gene Expression. Environ. Sci. Pollut. Res..

[61] Guo J., Shi L., Gong X., Jiang M., Yin Y., Zhang X., Yin H., Li H., Emori C., Sugiura K. (2016). Oocyte-Dependent Activation of MTOR in Cumulus Cells Controls the Development and Survival of Cumulus-Oocyte Complexes. J. Cell. Sci..

[62] Di Stefano L. (2003). E2F7, a Novel E2F Featuring DP-Independent Repression of a Subset of E2F-Regulated Genes. EMBO J..

[63] Shi J., Yoshino O., Osuga Y., Akiyama I., Harada M., Koga K., Fujimoto A., Yano T., Taketani Y. (2012). Growth Differentiation Factor 3 Is Induced by Bone Morphogenetic Protein 6 (BMP-6) and BMP-7 and Increases Luteinizing Hormone Receptor Messenger RNA Expression in Human Granulosa Cells. Fertil. Steril..

[64] Spitschak M., Hoeflich A. (2018). Potential Functions of IGFBP-2 for Ovarian Folliculogenesis and Steroidogenesis. Front. Endocrinol..

[65] Maffi A.S., Tonellotto Dos Santos J., Caetano De Oliveira F., Gasperin B.G., Schneider A., Rincón J.A.A., Rabassa V.R., Burkert Del Pino F.A., Corrêa M.N., Brauner C.C. (2019). Insulin Treatment Does Not Affect Follicular Development but Alters Granulosa Cell Gene Expression in Dairy Cows. Theriogenology.

[66] Mazerbourg S., Monget P. (2018). Insulin-Like Growth Factor Binding Proteins and IGFBP Proteases: A Dynamic System Regulating the Ovarian Folliculogenesis. Front. Endocrinol..

[67] Satrapa R.A., Castilho A.S., Razza E.M., Pegorer M.F., Puelker R., Barros C.M. (2013). Differential Expression of Members of the IGF System in OPU-Derived Oocytes from Nelore (Bos Indicus) and Holstein (Bos Taurus) Cows. Anim. Reprod. Sci..

[68] Nuttinck F., Charpigny G., Mermillod P., Loosfelt H., Meduri G., Freret S., Grimard B., Heyman Y. (2004). Expression of Components of the Insulin-like Growth Factor System and Gonadotropin Receptors in Bovine Cumulus–Oocyte Complexes during Oocyte Maturation. Domest. Anim. Endocrinol..

[69] Regassa A., Rings F., Hoelker M., Cinar U., Tholen E., Looft C., Schellander K., Tesfaye D. (2011). Transcriptome Dynamics and Molecular Cross-Talk between Bovine Oocyte and Its Companion Cumulus Cells. BMC Genom..

[70] Kulus M., Kranc W., Sujka-Kordowska P., Mozdziak P., Jankowski M., Konwerska A., Kulus J., Bukowska D., Skowroński M., Piotrowska-Kempisty H. (2020). The Processes of Cellular Growth, Aging, and Programmed Cell Death Are Involved in Lifespan of Ovarian Granulosa Cells during Short-Term IVC—Study Based on Animal Model. Theriogenology.

[71] Brązert M., Kranc W., Nawrocki M., Sujka-Kordowska P., Konwerska A., Jankowski M., Kocherova I., Celichowski P., Jeseta M., Ożegowska K. (2020). New Markers for Regulation of Transcription and Macromolecule Metabolic Process in Porcine Oocytes during in Vitro Maturation. Mol. Med. Rep..

[72] Li Y., Xiong G., Tan J., Wang S., Wu Q., Wan L., Zhang Z., Huang O. (2021). Aberrant Activation of the Hedgehog Signaling Pathway in Granulosa Cells from Patients with Polycystic Ovary Syndrome. Bioengineered.

[73] Liu C., Peng J., Matzuk M.M., Yao H.H.-C. (2015). Lineage Specification of Ovarian Theca Cells Requires Multicellular Interactions via Oocyte and Granulosa Cells. Nat. Commun..

[74] Liu Y., Li Z., Wang Y., Cai Q., Liu H., Xu C., Zhang F. (2022). IL-15 Participates in the Pathogenesis of Polycystic Ovary Syndrome by Affecting the Activity of Granulosa Cells. Front. Endocrinol..

[75] Machlin J.H., Barishansky S.J., Kelsh J., Larmore M.J., Johnson B.W., Pritchard M.T., Pavone M.E., Duncan F.E. (2021). Fibroinflammatory Signatures Increase with Age in the Human Ovary and Follicular Fluid. Int. J. Mol. Sci..

[76] Kaur S., Archer K.J., Devi M.G., Kriplani A., Strauss J.F., Singh R. (2012). Differential Gene Expression in Granulosa Cells from Polycystic Ovary Syndrome Patients with and without Insulin Resistance: Identification of Susceptibility Gene Sets through Network Analysis. J. Clin. Endocrinol. Metab..

[77] Hatzirodos N., Hummitzsch K., Irving-Rodgers H.F., Rodgers R.J. (2015). Transcriptome Comparisons Identify New Cell Markers for Theca Interna and Granulosa Cells from Small and Large Antral Ovarian Follicles. PLoS ONE.

[78] Nore A., Juarez-Martinez A.B., Clément J., Brun C., Diagouraga B., Laroussi H., Grey C., Bourbon H.M., Kadlec J., Robert T. (2022). TOPOVIBL-REC114 Interaction Regulates Meiotic DNA Double-Strand Breaks. Nat. Commun..

[79] Jo M., Curry T.E. (2006). Luteinizing Hormone-Induced RUNX1 Regulates the Expression of Genes in Granulosa Cells of Rat Periovulatory Follicles. J. Mol. Endocrinol..

[80] Abe T., Lee A., Sitharam R., Kesner J., Rabadan R., Shapira S.D. (2017). Germ-Cell-Specific Inflammasome Component NLRP14 Negatively Regulates Cytosolic Nucleic Acid Sensing to Promote Fertilization. Immunity.

[81] Dankert D., Demond H., Trapphoff T., Heiligentag M., Rademacher K., Eichenlaub-Ritter U., Horsthemke B., Grümmer R. (2014). Pre- and Postovulatory Aging of Murine Oocytes Affect the Transcript Level and Poly(A) Tail Length of Maternal Effect Genes. PLoS ONE.

[82] Molinari E., Bar H., Pyle A.M., Patrizio P. (2016). Transcriptome Analysis of Human Cumulus Cells Reveals Hypoxia as the Main Determinant of Follicular Senescence. Mol. Hum. Reprod..

[83] Bergandi L., Basso G., Evangelista F., Canosa S., Dalmasso P., Aldieri E., Revelli A., Benedetto C., Ghigo D. (2014). Inducible Nitric Oxide Synthase and Heme Oxygenase 1 Are Expressed in Human Cumulus Cells and May Be Used as Biomarkers of Oocyte Competence. Reprod. Sci..

[84] Sammad A., Luo H., Hu L., Zhu H., Wang Y. (2022). Transcriptome Reveals Granulosa Cells Coping through Redox, Inflammatory and Metabolic Mechanisms under Acute Heat Stress. Cells.

[85] Huo L.-J., Liang C.-G., Yu L.-Z., Zhong Z.-S., Yang Z.-M., Fan H.-Y., Chen D.-Y., Sun Q.-Y. (2005). Inducible Nitric Oxide Synthase-Derived Nitric Oxide Regulates Germinal Vesicle Breakdown and First Polar Body Emission in the Mouse Oocyte. Reproduction.

[86] Nath P., Maitra S. (2019). Physiological Relevance of Nitric Oxide in Ovarian Functions: An Overview. Gen. Comp. Endocrinol..

[87] Nanda N., Bao M., Lin H., Clauser K., Komuves L., Quertermous T., Conley P.B., Phillips D.R., Hart M.J. (2005). Platelet Endothelial Aggregation Receptor 1 (PEAR1), a Novel Epidermal Growth Factor Repeat-Containing Transmembrane Receptor, Participates in Platelet Contact-Induced Activation. J. Biol. Chem..

[88] Chiba H., Michibata H., Wakimoto K., Seishima M., Kawasaki S., Okubo K., Mitsui H., Torii H., Imai Y. (2004). Cloning of a Gene for a Novel Epithelium-Specific Cytosolic Phospholipase A2, CPLA2δ, Induced in Psoriatic Skin. J. Biol. Chem..

[89] Yang H., Lin S., Lei X., Yuan C., Tian Z., Yu Y., Zhao Z., Chen J. (2016). Identification and Profiling of MicroRNAs from Ovary of Estrous Kazakh Sheep Induced by Nutritional Status in the Anestrous Season. Anim. Reprod. Sci..

[90] Zhou H., Xu Q.-Z., Zhang R., Zhuang Z.-X., Ma Y.-Q., Wang W., Ma T.-Y., Sui Y., Liu Y., Cao X. (2018). Gonadal Transcriptome Analysis of Hybrid Triploid Loaches (*Misgurnus anguillicaudatus*) and Their Diploid and Tetraploid Parents. PLoS ONE.

[91] Shrestha K., Al-Alem L., Garcia P., Wynn M.A.A., Hannon P.R., Jo M., Drnevich J., Duffy D.M., Curry T.E. (2022). Neurotensin Expression, Regulation, and Function during the Ovulatory Period in the Mouse Ovary. Biol. Reprod..

[92] Cox L., Vanderwall D.K., Parkinson K.C., Sweat A., Isom S.C. (2015). Expression Profiles of Select Genes in Cumulus–Oocyte Complexes from Young and Aged Mares. Reprod. Fertil. Dev..

[93] Yang X., Dunning K.R., Wu L.L.-Y., Hickey T.E., Norman R.J., Russell D.L., Liang X., Robker R.L. (2010). Identification of Perilipin-2 as a Lipid Droplet Protein Regulated in Oocytes during Maturation. Reprod. Fertil. Dev..

[94] Huang J.Z., Huang L.M., Zeng Q.J., Huang E.F., Liang H.P., Wei Q., Xie X.H., Ruan J.M. (2018). Distribution and Quantitative Analysis of *CIDEa* and *CIDEc* in Broiler Chickens: Accounting for Differential Fat Deposition between Strains. Br. Poult. Sci..

[95] Congras A., Yerle-Bouissou M., Pinton A., Vignoles F., Liaubet L., Ferchaud S., Acloque H. (2014). Sperm DNA Methylation Analysis in Swine Reveals Conserved and Species-Specific Methylation Patterns and Highlights an Altered Methylation at the GNAS Locus in Infertile Boars1. Biol. Reprod..

[96] Lu X., Abdalla I.M., Nazar M., Fan Y., Zhang Z., Wu X., Xu T., Yang Z. (2021). Genome-Wide Association Study on Reproduction-Related Body-Shape Traits of Chinese Holstein Cows. Animals.

[97] Dias M.M., Cánovas A., Mantilla-Rojas C., Riley D.G., Luna-Nevarez P., Coleman S.J., Speidel S.E., Enns R.M., Islas-Trejo A., Medrano J.F. (2017). SNP Detection Using RNA-Sequences of Candidate Genes Associated with Puberty in Cattle. Genet. Mol. Res..

[98] Li Y., Zhang Y., He B., Wang Y., Yuan Z., Yuan W., Liao P., Deng Y., Xiao J., Zhu C. (2007). Cloning and Expression of a Novel Human Gene, Isl-2, Encoded a LIM-Homeodomain Protein. Mol. Biol. Rep..

[99] Mallya M., Campbell R.D., Aguado B. (2002). Transcriptional Analysis of a Novel Cluster of LY-6 Family Members in the Human and Mouse Major Histocompatibility Complex: Five Genes with Many Splice Forms. Genomics.

[100] Choudhury A., Khole V. (2015). Immune-Mediated Destruction of Ovarian Follicles Associated with the Presence of HSP90 Antibodies. Mol. Reprod. Dev..

[101] Etchevers L., Stassi A.F., Belotti E.M., Diaz P.U., Durante L.I., Notaro U.S., Chiaraviglio J.A., Rey F., Salvetti N.R., Ortega H.H. (2023). Exogenous ACTH Stimulus during the Preovulatory Period Alters Patterns of Leukocyte Recruitment in the Ovary of Dairy Cows. Theriogenology.

[102] Atli M.O., Mehta V., Vezina C.M., Wiltbank M.C. (2022). Expression Patterns of Chemokine (C–C Motif) Ligand 2, Prostaglandin F2A Receptor and Immediate Early Genes at MRNA Level in the Bovine Corpus Luteum after Intrauterine Treatment with a Low Dose of Prostaglandin F2A. Theriogenology.

[103] Zhang W., Fu Q., Yao K. (2020). A Three-mRNA Status Risk Score Has Greater Predictive Ability Compared with a LncRNA-based Risk Score for Predicting Prognosis in Patients with Hepatocellular Carcinoma. Oncol. Lett..

[104] Li Z., Zhang X., Xie S., Liu X., Fei C., Huang X., Tang Y., Zhou L. (2022). H3K36me2 Methyltransferase NSD2 Orchestrates Epigenetic Reprogramming during Spermatogenesis. Nucleic Acids Res..

[105] Dong L., Xin X., Chang H.-M., Leung P.C.K., Yu C., Lian F., Wu H. (2022). Expression of Long Noncoding RNAs in the Ovarian Granulosa Cells of Women with Diminished Ovarian Reserve Using High-Throughput Sequencing. J. Ovarian Res..

[106] Yang G.-P., He W.-P., Tan J.-F., Yang Z.-X., Fan R.-R., Ma N.-F., Wang F.-W., Chen L., Li Y., Shen H.-W. (2019). Overexpression of SLC12A5 Is Associated with Tumor Progression and Poor Survival in Ovarian Carcinoma. Int. J. Gynecol. Canc..

[107] Fábryová K., Simon M. (2009). Function of the Cell Surface Molecules (CD Molecules) in the Reproduction Processes. Gen. Physiol. Biophys..

[108] Mahdipour M., Van Tol H.T.A., Stout T.A.E., Roelen B.A.J. (2015). Validating Reference MicroRNAs for Normalizing QRT-PCR Data in Bovine Oocytes and Preimplantation Embryos. BMC Dev. Biol..

[109] Ren J., Hao Y., Liu Z., Li S., Wang C., Wang B., Liu Y., Liu G., Dai Y. (2021). Effect of Exogenous Glutathione Supplementation on the in Vitro Developmental Competence of Ovine Oocytes. Theriogenology.

[110] Latham K.E., Kutyna K., Wang Q. (1999). Genetic Variation in Trophectoderm Function in Parthenogenetic Mouse Embryos. Dev. Genet..

[111] Laitinen M., Vuojolainen K., Jaatinen R., Ketola I., Aaltonen J., Lehtonen E., Heikinheimo M., Ritvos O. (1998). A Novel Growth Differentiation Factor-9 (GDF-9) Related Factor Is Co-Expressed with GDF-9 in Mouse Oocytes during Folliculogenesis. Mech. Dev..

[112] Galloway S.M., McNatty K.P., Cambridge L.M., Laitinen M.P.E., Juengel J.L., Jokiranta T.S., McLaren R.J., Luiro K., Dodds K.G., Montgomery G.W. (2000). Mutations in an Oocyte-Derived Growth Factor Gene (BMP15) Cause Increased Ovulation Rate and Infertility in a Dosage-Sensitive Manner. Nat. Genet..

[113] Gilchrist R.B., Ritter L.J., Cranfield M., Jeffery L.A., Amato F., Scott S.J., Myllymaa S., Kaivo-Oja N., Lankinen H., Mottershead D.G. (2004). Immunoneutralization of Growth Differentiation Factor 9 Reveals It Partially Accounts for Mouse Oocyte Mitogenic Activity1. Biol. Reprod..

[114] Cecconi S., Ciccarelli C., Barberi M., Macchiarelli G., Canipari R. (2004). Granulosa Cell-Oocyte Interactions. Eur. J. Obstet. Gynecol. Reprod. Biol..

[115] Cai Q., Yan J., Duan R., Zhu Y., Hua Y., Liao Y., Li Q., Li W., Ji S. (2023). E3 Ligase Cul2 Mediates Drosophila Early Germ Cell Differentiation through Targeting Bam. Dev. Biol..

[116] Douville G., Sirard M.-A. (2014). Changes in Granulosa Cells Gene Expression Associated with Growth, Plateau and Atretic Phases in Medium Bovine Follicles. J. Ovarian Res..

[117] Liu M., Hummitzsch K., Bastian N.A., Hartanti M.D., Wan Q., Irving-Rodgers H.F., Anderson R.A., Rodgers R.J. (2022). Isolation, Culture, and Characterisation of Bovine Ovarian Fetal Fibroblasts and Gonadal Ridge Epithelial-like Cells and Comparison to Their Adult Counterparts. PLoS ONE.

[118] Xie H., Xu H., Hou Y., Cai Y., Rong Z., Song W., Wang W., Li K. (2019). Integrative Prognostic Subtype Discovery in High-grade Serous Ovarian Cancer. J. Cell. Biochem..

[119] Maraldi T., Resca E., Nicoli A., Beretti F., Zavatti M., Capodanno F., Morini D., Palomba S., la Sala G.B., de Pol A. (2016). NADPH Oxidase-4 and MATER Expressions in Granulosa Cells: Relationships with Ovarian Aging. Life Sci..

[120] Huang X., Sun Q., Chen D., Yang W., Zhang J., Liu R., Zhang P., Huang L., Zhang M., Fu Q. (2022). *Nlrp5* and *Tle6* Expression Patterns in Buffalo Oocytes and Pre-implantation Embryos. Reprod. Domest. Anim..

[121] Bebbere D., Abazari-Kia A., Nieddu S., Melis Murgia B., Albertini D.F., Ledda S. (2020). Subcortical Maternal Complex (SCMC) Expression during Folliculogenesis Is Affected by Oocyte Donor Age in Sheep. J. Assist. Reprod. Genet..

[122] Tong Z.-B., Gold L., De Pol A., Vanevski K., Dorward H., Sena P., Palumbo C., Bondy C.A., Nelson L.M. (2004). Developmental Expression and Subcellular Localization of Mouse MATER, an Oocyte-Specific Protein Essential for Early Development. Endocrinology.

[123] Tong Z.-B., Gold L., Pfeifer K.E., Dorward H., Lee E., Bondy C.A., Dean J., Nelson L.M. (2000). Mater, a Maternal Effect Gene Required for Early Embryonic Development in Mice. Nat. Genet..

[124] Tong Z.-B., Nelson L.M., Dean J. (2000). Mater Encodes a Maternal Protein in Mice with a Leucine-Rich Repeat Domain Homologous to Porcine Ribonuclease Inhibitor. Mamm. Genome.

[125] Sena P., Riccio M., Marzona L., Nicoli A., Marsella T., Marmiroli S., Bertacchini J., Fano R.A., La Sala G.B., de Pol A. (2009). Human MATER Localization in Specific Cell Domains of Oocytes and Follicular Cells. Reprod. Biomed. Online.

[126] Summers A.F., Pohlmeier W.E., Sargent K.M., Cole B.D., Vinton R.J., Kurz S.G., McFee R.M., Cushman R.A., Cupp A.S., Wood J.R. (2014). Altered Theca and Cumulus Oocyte Complex Gene Expression, Follicular Arrest and Reduced Fertility in Cows with Dominant Follicle Follicular Fluid Androgen Excess. PLoS ONE.

[127] Kim A.M., Vogt S., O’Halloran T., Woodruff T.K. (2010). Zinc Availability Regulates Exit from Meiosis in Maturing Mammalian Oocytes. Nat. Chem. Biol..

[128] Bernhardt M.L., Kim A.M., O’Halloran T., Woodruff T.K. (2011). Zinc Requirement During Meiosis I–Meiosis II Transition in Mouse Oocytes Is Independent of the MOS-MAPK Pathway1. Biol. Reprod..

[129] Suzuki T., Yoshida N., Suzuki E., Okuda E., Perry A.C.F. (2010). Full-Term Mouse Development by Abolishing Zn2+-Dependent Metaphase II Arrest without Ca2+ Release. Development.

[130] Kim A.M., Bernhardt M.L., Kong B.Y., Ahn R.W., Vogt S., Woodruff T.K., O’Halloran T.V. (2011). Zinc Sparks Are Triggered by Fertilization and Facilitate Cell Cycle Resumption in Mammalian Eggs. ACS Chem. Biol..

[131] Lisle R.S., Anthony K., Randall M.A., Diaz F.J. (2013). Oocyte–Cumulus Cell Interactions Regulate Free Intracellular Zinc in Mouse Oocytes. Reproduction.

[132] Oh J.S., Han S.J., Conti M. (2010). Wee1B, Myt1, and Cdc25 Function in Distinct Compartments of the Mouse Oocyte to Control Meiotic Resumption. J. Cell. Biol..

[133] Solc P., Schultz R.M., Motlik J. (2010). Prophase I Arrest and Progression to Metaphase I in Mouse Oocytes: Comparison of Resumption of Meiosis and Recovery from G2-Arrest in Somatic Cells. Mol. Hum. Reprod..

[134] Bonnet A., Servin B., Mulsant P., Mandon-Pepin B. (2015). Spatio-Temporal Gene Expression Profiling during In Vivo Early Ovarian Folliculogenesis: Integrated Transcriptomic Study and Molecular Signature of Early Follicular Growth. PLoS ONE.

[135] Han S.J., Chen R., Paronetto M.P., Conti M. (2005). Wee1B Is an Oocyte-Specific Kinase Involved in the Control of Meiotic Arrest in the Mouse. Curr. Biol..

[136] Hanna C.B., Yao S., Patta M.C., Jensen J.T., Wu X. (2010). WEE2 Is an Oocyte-Specific Meiosis Inhibitor in Rhesus Macaque Monkeys1. Biol. Reprod..

[137] Zhang Z., Mu J., Zhao J., Zhou Z., Chen B., Wu L., Yan Z., Wang W., Zhao L., Dong J. (2019). Novel Mutations in *WEE2*: Expanding the Spectrum of Mutations Responsible for Human Fertilization Failure. Clin. Genet..

[138] Mitwally M.F., Casper R.F., Diamond M.P. (2005). The Role of Aromatase Inhibitors in Ameliorating Deleterious Effects of Ovarian Stimulation on Outcome of Infertility Treatment. Reprod. Biol. Endocrinol..

[139] Li Z., Li T., Leng Y., Chen S., Liu Q., Feng J., Chen H., Huang Y., Zhang Q. (2018). Hormonal Changes and Folliculogenesis in Female Offspring of Rats Exposed to Cadmium during Gestation and Lactation. Environ. Pollut..

[140] Das S., Mukherjee D. (2013). Effect of Cadmium Chloride on Secretion of 17β-Estradiol by the Ovarian Follicles of Common Carp, Cyprinus Carpio. Gen. Comp. Endocrinol..

[141] Zhou J., Wang J., Penny D., Monget P., Arraztoa J.A., Fogelson L.J., Bondy C.A. (2003). Insulin-like Growth Factor Binding Protein 4 Expression Parallels Luteinizing Hormone Receptor Expression and Follicular Luteinization in the Primate Ovary. Biol. Reprod..

[142] Ramirez D.C., Martinez L.D., Marchevsky E., Gimenez M.S. (1999). Biphasic Effect of Cadmium in Non-Cytotoxic Conditions on the Secretion of Nitric Oxide from Peritoneal Macrophages. Toxicology.

[143] Sangartit W., Kukongviriyapan U., Donpunha W., Pakdeechote P., Kukongviriyapan V., Surawattanawan P., Greenwald S.E. (2014). Tetrahydrocurcumin Protects against Cadmium-Induced Hypertension, Raised Arterial Stiffness and Vascular Remodeling in Mice. PLoS ONE.

[144] Refaie M.M.M., El-Hussieny M., Zenhom N.M. (2018). Protective Role of Nebivolol in Cadmium-Induced Hepatotoxicity via Downregulation of Oxidative Stress, Apoptosis and Inflammatory Pathways. Environ. Toxicol. Pharmacol..

[145] Fouad A.A., Qureshi H.A., Al-Sultan A.I., Yacoubi M.T., Ali A.A. (2009). Protective Effect of Hemin against Cadmium-Induced Testicular Damage in Rats. Toxicology.

[146] Van Voorhis B.J., Dunn M.S., Snyder G.D., Weiner C.P. (1994). Nitric Oxide: An Autocrine Regulator of Human Granulosa-Luteal Cell Steroidogenesis. Endocrinology.

[147] Zhang T., Zhang C., Zhang J., Lin J., Song D., Zhang P., Liu Y., Chen L., Zhang L. (2022). Cadmium Impairs Zebrafish Swim Bladder Development via ROS Mediated Inhibition of the Wnt/Hedgehog Pathway. Aquat. Toxicol..

[148] Sabolić I., Breljak D., Skarica M., Herak-Kramberger C.M. (2010). Role of Metallothionein in Cadmium Traffic and Toxicity in Kidneys and Other Mammalian Organs. Biometals.

[149] Liu Y.P., Liu J., Iszard M.B., Andrews G.K., Palmiter R.D., Klaassen C.D. (1995). Transgenic Mice That Overexpress Metallothionein-I Are Protected from Cadmium Lethality and Hepatotoxicity. Toxicol. Appl. Pharmacol..

[150] Shen X., Liu W., Chen Y., Guo Y., Gao M., Chen W., Liu Y., Liu S. (2019). Diagnostic Significance of Metallothionein Members in Recognizing Cadmium Exposure in Various Organs under Low-Dose Exposure. Chemosphere.

[151] Kluxen F.M., Höfer N., Kretzschmar G., Degen G.H., Diel P. (2012). Cadmium Modulates Expression of Aryl Hydrocarbon Receptor-Associated Genes in Rat Uterus by Interaction with the Estrogen Receptor. Arch. Toxicol..

[152] Bridges C.C., Zalups R.K. (2005). Molecular and Ionic Mimicry and the Transport of Toxic Metals. Toxicol. Appl. Pharmacol..

[153] Forti E., Bulgheroni A., Cetin Y., Hartung T., Jennings P., Pfaller W., Prieto P. (2010). Characterisation of Cadmium Chloride Induced Molecular and Functional Alterations in Airway Epithelial Cells. Cell Physiol. Biochem..

[154] Han S.G., Castranova V., Vallyathan V. (2007). Comparative Cytotoxicity of Cadmium and Mercury in a Human Bronchial Epithelial Cell Line (BEAS-2B) and Its Role in Oxidative Stress and Induction of Heat Shock Protein 70∗. J. Toxicol. Environ. Health A.

[155] Hofmann U., Priem M., Bartzsch C., Winckler T., Feller K.-H. (2014). A Sensitive Sensor Cell Line for the Detection of Oxidative Stress Responses in Cultured Human Keratinocytes. Sensors.

[156] Wada K.-I., Taniguchi A., Okano T. (2007). Highly Sensitive Detection of Cytotoxicity Using a Modified HSP70B′ Promoter. Biotechnol. Bioeng..

[157] Singh P., Chandrasekaran V., Hardy B., Wilmes A., Jennings P., Exner T.E. (2021). Temporal Transcriptomic Alterations of Cadmium Exposed Human IPSC-Derived Renal Proximal Tubule-like Cells. Toxicol. Vitr..

[158] Wang W., Liu G., Jiang X., Wu G. (2021). Resveratrol Ameliorates Toxic Effects of Cadmium on Placental Development in Mouse Placenta and Human Trophoblast Cells. Birth Defects Res..

[159] Park M.J., Ahn J.-W., Kim K.H., Bang J., Kim S.C., Jeong J.Y., Choi Y.E., Kim C.-W., Joo B.S. (2020). Prediction of Ovarian Aging Using Ovarian Expression of BMP15, GDF9, and C-KIT. Exp. Biol. Med..

[160] Daneshjou D., Soleimani Mehranjani M., Zadeh Modarres S., Shariatzadeh M.A. (2020). Sitagliptin/Metformin: A New Medical Treatment in Polycystic Ovary Syndrome. Trends Endocrinol. Metab..

[161] Lu M., Fu D. (2007). Structure of the Zinc Transporter YiiP. Science.

[162] Chemek M., Boughammoura S., Ben Mimouna S., Chouchene L., Banni M., Messaoudi I. (2015). Changes of the MRNA Expression Pattern of Zn Transporters: A Probable Mechanism for Cadmium Retention and Zinc Redistribution in the Suckling Rat Tissues. Biol. Trace Elem. Res..

