# Supplementary material for: Cumulus Cell Transcriptome after Cumulus-Oocyte Complex Exposure to Nanomolar Cadmium in an In Vitro Animal Model of Prepubertal and Adult Age
Source: Biology (Basel). 2023 Feb 4;12(2):249. doi: 10.3390/biology12020249 (PMC9953098; doi:10.3390/biology12020249)
Supplement: Supplementary file 1 [file biology-12-00249-s001.zip › Table S1 - Martino R1.pdf]

Table S1: Upregulated genes in CCs of adult sheep exposed in vitro to nanomolar cadmium

Gene ID and description	Log2	P value	Gene Cards (*) (**) (§)	PubMed data found with key words: Cumulus, Granulosa, Oocyte, Ovary	References
**ABCC8**<br><br>ATP binding cassette Subfamily C member 8	2,26	0,004	The protein encoded by this gene is a member of the superfamily of ATP-binding cassette (ABC) transporters. ABC proteins transport various molecules across extra- and intra-cellular membranes. ABC genes are divided into seven distinct subfamilies (ABC1, MDR/TAP, MRP, ALD, OABP, GCN20, White). This protein is a member of the MRP subfamily which is involved in multi-drug resistance. This protein functions as a modulator of ATP-sensitive potassium channels and insulin release. Mutations in the ABCC8 gene and deficiencies in the encoded protein have been observed in patients with hyperinsulinemic hypoglycemia of infancy, an autosomal recessive disorder of unregulated and high insulin secretion. Mutations have also been associated with non-insulin-dependent diabetes mellitus type II, an autosomal dominant disease of defective insulin secretion. [RefSeq, Jul 2020] (**)		
**ACCSL**<br><br>Aminocyclopropane-1-Carboxylate Synthase Homolog (Inactive) Like	4,31	0,003	Predicted to enable catalytic activity and pyridoxal phosphate binding activity. Predicted to be involved in biosynthetic process. [Alliance of Genome Resources, Apr 2022] (**)		
**ADGRF4**<br><br>Adhesion G Protein-Coupled Receptor F4	1,71	0,023	Sequence analysis of this gene suggests that it is encodes a member of the superfamily of G protein-couple receptors. G protein-coupled receptors typically contain seven hydrophobic transmembrane domains, interact with guanine nucleotide binding regulatory proteins, and detect molecules outside the cell and act to transduce these signals into intracellular responses. [RefSeq, Dec 2016] (**)		
**APOA2**<br><br>Apolipoprotein A2	2,93	0,002	This gene encodes apolipoprotein (apo-) A-II, which is the second most abundant protein of the high density lipoprotein particles. The protein is found in plasma as a monomer, homodimer, or heterodimer with apolipoprotein D. Defects in this gene may result in apolipoprotein A-II deficiency or hypercholesterolemia. [RefSeq, Jul 2008] (**) (§)		
**ATAD5**<br><br>ATPase Family AAA Domain Containing 5	1,23	0,001	Enables DNA clamp unloader activity. Involved in DNA clamp unloading; positive regulation of DNA replication; and positive regulation of cell cycle G2/M phase transition. Part of Elg1 RFC-like complex. Biomarker of neurilemmoma.<br>[Alliance of Genome Resources, Apr 2022] (**)		
**CCNE2**<br><br>Cyclin E2	1,24	0,004	The protein encoded by this gene belongs to the highly conserved cyclin family, whose members are characterized by a dramatic periodicity in protein abundance through the cell cycle. Cyclins function as regulators of CDK kinases. Different cyclins exhibit distinct expression and degradation patterns which contribute to the temporal coordination of each mitotic event. This cyclin forms a complex with and functions as a regulatory subunit of CDK2. This cyclin has been shown to specifically interact with CIP/KIP family of CDK inhibitors, and plays a role in cell cycle G1/S transition. The expression of this gene peaks at the G1-S phase and exhibits a pattern of tissue specificity distinct from that of cyclin E1. A significantly increased expression level of this gene was observed in tumor-derived cells. [RefSeq, Jul 2008] (**)	DNA replication and cell cycle progression in COCs	[51]
**CD274**<br><br>CD274 Molecule	1,76	0,031	This gene encodes an immune inhibitory receptor ligand that is expressed by hematopoietic and non-hematopoietic cells, such as T cells and B cells and various types of tumor cells. The encoded protein is a type I transmembrane protein that has immunoglobulin V-like and C-like domains. Interaction of this ligand with its receptor inhibits T-cell activation and cytokine production. During infection or inflammation of normal tissue, this interaction is important for preventing autoimmunity by maintaining homeostasis of the immune response. In tumor microenvironments, this interaction provides an immune escape for tumor cells through cytotoxic		

			T-cell inactivation. Expression of this gene in tumor cells is considered to be prognostic in many types of human malignancies, including colon cancer and renal cell carcinoma. [RefSeq, Sep 2015] (**)		
**CDC6**<br><br>Cell Division Cycle 6	1,69	0,001	The protein encoded by this gene is highly similar to Saccharomyces cerevisiae Cdc6, a protein essential for the initiation of DNA replication. This protein functions as a regulator at the early steps of DNA replication. It localizes in cell nucleus during cell cyle G1,but translocates to the cytoplasm at the start of S phase. The subcellular translocation of this protein during cell cyle is regulated through its phosphorylation by Cdks. Transcription of this protein was reported to be regulated in response to mitogenic signals through transcriptional control mechanism involving E2F proteins. [RefSeq, Jul 2008] (**)	CDC6 regulates both G2/M transition and metaphase-to-anaphase transition during the first meiosis of mouse oocytes	[52]
				It is essential for spindle formation in mouse oocytes	[53]
**CENPK**<br><br>Centromere Protein K	1,49	0,001	CENPK is a subunit of a CENPH (MIM 605607)-CENPI (MIM 300065)-associated centromeric complex that targets CENPA (MIM 117139) to centromeres and is required for proper kinetochore function and mitotic progression ([54][OMIM, Mar 2008] (**) (§)		
**CRISPLD1**<br><br>Cysteine Rich Secretory Protein LCCL Domain Containing 1	1,93	0,001	Involved in face morphogenesis. Located in extracellular exosome. [Alliance of Genome Resources, Apr 2022] (**)		
**CXCL14**<br><br>C-X-C Motif Chemokine Ligand 14	2,41	0,019	This antimicrobial gene belongs to the cytokine gene family which encode secreted proteins involved in immunoregulatory and inflammatory processes. The protein encoded by this gene is structurally related to the CXC (Cys-X-Cys) subfamily of cytokines. Members of this subfamily are characterized by two cysteines separated by a single amino acid. This cytokine displays chemotactic activity for monocytes but not for lymphocytes, dendritic cells, neutrophils or macrophages. It has been implicated that this cytokine is involved in the homeostasis of monocyte-derived macrophages rather than in inflammation. [RefSeq, Sep 2014] (**)	Lower expression of CXCL14 in human luteinized GCs resulted in the dysfunction of progesterone synthesis in PCOS patients.	[55]
				It has pro-inflammatory, vasodilatory, proteolytics and coagulatory functions in oocyte maturation and ovulation processes in the rainbow trout	[56]
**CYP19A1**<br><br>Cytochrome P450 Family 19 Subfamily A Member 1	4,09	0,002	This gene encodes a member of the cytochrome P450 superfamily of enzymes. The cytochrome P450 proteins are monooxygenases which catalyze many reactions involved in drug metabolism and synthesis of cholesterol, steroids and other lipids. This protein localizes to the endoplasmic reticulum and catalyzes the last steps of estrogen biosynthesis. Mutations in this gene can result in either increased or decreased aromatase activity; the associated phenotypes suggest that estrogen functions both as a sex steroid hormone and in growth or differentiation. [provided by RefSeq, Dec 2016] (**)	Transcriptome analysis of GCs of healthy follicles of sows with a high percentage high-quality COCs showed higher abundance of transcripts involved in ovarian steroidogenesis (e.g., CYP19A2 and 3, POR, VEGFA) and growth (IGF1)	[57]
				Disorders of the ovarian estrogen secretion are more likely to induce female estrogen-dependent diseases and fertility issues, such as ovarian cancer and PCOS. Hence, aromatase is an important drug target	[58]
				Upregulation of CYP19A1 in the hypoandrogenic PCOS group can validate the role of selected miRs in the prognosis of PCOS.	[59]
				It is a candidate susceptibility gene for PCOS and Infertility	[60]
				Bisphenol A alters the expression of various significant genes like GnRH, AdipoQ, ESR1, StAR, CYP11A1, CYP19A1, and many more involved in the pathways and endocrine regulation, whose disruption is commonly associated with the clinical manifestations of PCOS.	[61]
**DDIT4L**<br><br>DNA Damage Inducible Transcript 4 Like	1,80	0,005	Predicted to be involved in negative regulation of signal transduction. Predicted to be located in cytoplasm.<br>[Alliance of Genome Resources, Apr 2022] (**)	Suppression of Ddit4l expression allows for MTOR activation in CCs	[62]
**DLGAP1**<br><br>DLG Associated	1,35	0,027	Predicted to enable molecular adaptor activity. Predicted to be a structural constituent of postsynaptic density. Predicted to be involved in several processes,		

Protein 1			including aggresome assembly; regulation of postsynaptic neurotransmitter receptor activity; and regulation of proteasomal protein catabolic process. Predicted to be located in plasma membrane. Predicted to be part of postsynaptic density. Predicted to be active in glutamatergic synapse and postsynaptic density, intracellular component. [Alliance of Genome Resources, Apr 2022] (**)		
**DPP10**<br><br>Dipeptidyl Peptidase Like 10	1,27	0,001	This gene encodes a single-pass type II membrane protein that is a member of the S9B family in clan SC of the serine proteases. This protein has no detectable protease activity, most likely due to the absence of the conserved serine residue normally present in the catalytic domain of serine proteases. However, it does bind specific voltage-gated potassium channels and alters their expression and biophysical properties. Mutations in this gene have been associated with asthma. [RefSeq, Jul 2008] (**)		
**E2F**<br><br>E2F Transcription Factor 1	1,21	0,043	The protein encoded by this gene is a member of the E2F family of transcription factors. The E2F family plays a crucial role in the control of cell cycle and action of tumor suppressor proteins and is also a target of the transforming proteins of small DNA tumor viruses. The E2F proteins contain several evolutionally conserved domains found in most members of the family. These domains include a DNA binding domain, a dimerization domain which determines interaction with the differentiation regulated transcription factor proteins (DP), a transactivation domain enriched in acidic amino acids, and a tumor suppressor protein association domain which is embedded within the transactivation domain. This protein and another 2 members, E2F2 and E2F3, have an additional cyclin binding domain. This protein binds preferentially to retinoblastoma protein pRB in a cell-cycle dependent manner. It can mediate both cell proliferation and p53-dependent/independent apoptosis. [RefSeq, Jul 2008] (**)		
**E2F7**<br><br>E2F transcription factor 7	1,60	0,001	E2F transcription factors, such as E2F7, play an essential role in the regulation of cell cycle progression [63]). [OMIM, May 2008] (**)		
**ETNPPL**<br><br>Ethanolamine-Phosphate Phospho-Lyase	1,40	0,030	Enables ethanolamine-phosphate phospho-lyase activity. Predicted to be located in mitochondrial matrix.<br>[Alliance of Genome Resources, Apr 2022] (*)		
**FHAD1**<br><br>Forkhead Associated Phosphopeptide Binding Domain 1	1,32	0,006	FHAD1 is a Protein Coding gene. An important paralog of this gene is CCDC27 (**)		
**GDF3**<br><br>Growth Differentiation Factor 3	2,24	0,030	This gene encodes a secreted ligand of the TGF-beta (transforming growth factor-beta) superfamily of proteins. Ligands of this family bind various TGF-beta receptors leading to recruitment and activation of SMAD family transcription factors that regulate gene expression. The encoded preproprotein is proteolytically processed to generate each subunit of the disulfide-linked homodimer. This protein plays a role ocular and skeletal development. Mutations in this gene are associated with microphthalmia,coloboma, and skeletal abnormalities in human patients. [RefSeq, Aug 2016] (**)	It might work as a negative regulator of oocyte-derived BMP cytokines in the growing follicle	[64]
**IGFBP-2**<br><br>Insulin Growth Factor Binding Protein 2	2,59	0,001	The protein encoded by this gene is one of six similar proteins that bind insulin-like growth factors I and II (IGF-I and IGF-II). The encoded protein can be secreted into the bloodstream, where it binds IGF-I and IGF-II with high affinity, or it can remain intracellular, interacting with many different ligands. High expression levels of this protein promote the growth of several types of tumors and may be predictive of the chances of recovery of the patient. [RefSeq, Sep 2015] (**) (§)	IGFBP-2 has been shown to block FSH-dependent E2 production in the ovarian follicle. Involved in Folliculogenesis and Steroidogenesis.	[65]
				Decreases IGFBP2 in granulosa cells, has been correlated with positive follicle development.	[66]
				Atresia is characterized by an increase of IGFBP-2 and IGFBP-4 levels, leading to a decrease in IGF bioavailability.	[67]

				The greater bioavailability of IGF (low expression of IGFBPs) may contribute to improve oocyte quality and embryo development	[68]
				Its expression decreased in matured COCs	[69]
**IHH**<br><br>Indian Hedgehog Signaling Molecule	1,90	0,028	This gene encodes a member of the hedgehog family of proteins. The encoded preproprotein is proteolytically processed to generate multiple protein products, including an N-terminal fragment that is involved in signaling. Hedgehog family proteins are essential secreted signaling molecules that regulate a variety of developmental processes including growth, patterning and morphogenesis. The protein encoded by this gene specifically plays a role in bone growth and differentiation. Mutations in this gene are the cause of brachydactyly type A1, which is characterized by shortening or malformation of the fingers and toes. Mutations in this gene are also the cause of acrocapitofemoral dysplasia. [RefSeq, Nov 2015] (**)	Involved in protein metabolic processes of bovine CCs	[70]
				IHH is involved in processes of cellular growth, aging, and programmed cell death of GCs.	[71]
				It was downregulated after in vitro oocyte maturation, what might indicate decreased potential of the cell to develop in an environment different than physiological.	[72]
				Hedgehog signaling pathway (Hh) members, Ihh and Ptch2 were abnormally highly expressed in the PCOS tissue	[73]
				Ovaries lacking Dhh/Ihh exhibit theca layer loss, blunted steroid production, arrested folliculogenesis and failure to form corpora lutea	[74]
**IL15**<br><br>Interleukin 15	1,66	0,018	The protein encoded by this gene is a cytokine that regulates T and natural killer cell activation and proliferation. This cytokine and interleukine 2 share many biological activities. They are found to bind common hematopoietin receptor subunits, and may compete for the same receptor, and thus negatively regulate each other's activity. The number of CD8+ memory cells is shown to be controlled by a balance between this cytokine and IL2. This cytokine induces the activation of JAK kinases, as well as the phosphorylation and activation of transcription activators STAT3, STAT5, and STAT6. Studies of the mouse counterpart suggested that this cytokine may increase the expression of apoptosis inhibitor BCL2L1/BCL-x(L), possibly through the transcription activation activity of STAT6, and thus prevent apoptosis. [RefSeq, Feb 2011] (**)	The results indicate that IL-15 is involved in the pathogenesis of PCOS potentially by affecting survival, the inflammation state and steroidogenesis of granulosa cells	[75]
				Using a cytokine antibody array, we identified a unique fibroinflammatory cytokine signature in follicular fluid across an aging series of women (27.7-44.8 years). This signature (IL-3, IL-7, IL-15, TGFβ1, TGFβ3 and MIP-1) increased with chronologic age, was inversely correlated to anti-Müllerian hormone (AMH) levels, and was independent of body mass index (BMI)	[76]
**INHBE**<br>Inhibin Subunit Beta E	1,75	0,008	This gene encodes a member of the TGF-beta (transforming growth factor-beta) superfamily of proteins. The encoded preproprotein is proteolytically processed to generate an inhibin beta subunit. Inhibins have been implicated in regulating numerous cellular processes including cell proliferation, apoptosis, immune response and hormone secretion. This gene may be upregulated under conditions of endoplasmic reticulum stress, and this protein may inhibit cellular proliferation and growth in pancreas and liver. [RefSeq, Sep 2016] (**) (§)		
**ITGAX**<br><br>Integrin Subunit Alpha X	1,51	0,047	This gene encodes the integrin alpha X chain protein. Integrins are heterodimeric integral membrane proteins composed of an alpha chain and a beta chain. This protein combines with the beta 2 chain (ITGB2) to form a leukocyte-specific integrin referred to as inactivated-C3b (iC3b) receptor 4 (CR4). The alpha X beta 2 complex seems to overlap the properties of the alpha M beta 2 integrin in the adherence of neutrophils and monocytes to stimulated endothelium cells, and in the phagocytosis of complement coated particles. Two transcript variants encoding different isoforms have been found for this gene. [RefSeq, Nov 2013] (**) (§)	Increased expression of inflammation genes ITGAX (3.68-fold) and TAB2 (1.86-fold) was confirmed in PCOS patients without insulin resistance	[77]
**KCNE3**<br><br>Potassium Potassium Voltage-Gated Channel Subfamily E Regulatory Subunit 3	1,53	0,008	Voltage-gated potassium (Kv) channels represent the most complex class of voltage-gated ion channels from both functional and structural standpoints. Their diverse functions include regulating neurotransmitter release, heart rate, insulin secretion, neuronal excitability, epithelial electrolyte transport, smooth muscle contraction, and cell volume. This gene encodes a member of the potassium channel, voltage-gated, isk-related subfamily. This member is a type I membrane protein, and a beta subunit that assembles with a potassium channel alpha-subunit to modulate the gating kinetics and enhance stability of the		

			multimeric complex. This gene is prominently expressed in the kidney. A missense mutation in this gene is associated with hypokalemic periodic paralysis. [RefSeq, Jul 2008] (**)		
**LOXL1**<br><br>Lysysl oxidase like 1	1,53	0,006	This gene encodes a member of the lysyl oxidase family of proteins. The prototypic member of the family is essential to the biogenesis of connective tissue, encoding an extracellular copper-dependent amine oxidase that catalyzes the first step in the formation of crosslinks in collagen and elastin. The encoded preproprotein is proteolytically processed to generate the mature enzyme. (**)	It was up regulated in the theca interna encoding for enzymes that process extracellular matrix.	[78]
**MEI1**<br><br>Meiotic Double-Stranded Break Formation Protein 1	1,20	0,035	Predicted to be involved in meiosis I. Predicted to act upstream of or within gamete generation; meiotic spindle organization; and meiotic telomere clustering. Implicated in gestational trophoblastic neoplasm. [Alliance of Genome Resources, Apr 2022] (**)	It regulates meiotic DNA double-strand breaks. Referred to spermatocytes and oocytes.	[79]
**MT1A**<br><br>Metallothionein 1A	1,28	0,001	This gene is a member of the metallothionein family of genes. Proteins encoded by this gene family are low in molecular weight, are cysteine-rich, lack aromatic residues, and bind divalent heavy metal ions. The conserved cysteine residues co-ordinate metal ions using mercaptide linkages. These proteins act as anti-oxidants, protect against hydroxyl free radicals, are important in homeostatic control of metal in the cell, and play a role in detoxification of heavy metals. Disruption of two metallothionein genes in mouse resulted in defects in protection against heavy metals, oxidative stress, immune reactions, carcinogens, and displayed obesity. [RefSeq, Sep 2017] (**)	Knockdown of Runx1 mRNA by small interfering RNA decreased progesterone secretion and reduced levels of mRNA for Cyp11a1, Hapln1, Mt1a, and Rgc32. The hormonally regulated expression of Runx1 in periovulatory follicles, its involvement in progesterone production, and regulation of preovulatory gene expression suggest important roles of RUNX1 in the periovulatory process.	[80]
**MYO5C**<br><br>Myosin VC	1,40	0,025	Predicted to enable actin filament binding activity and microfilament motor activity. Predicted to be involved in actin filament organization and vesicle transport along actin filament. Located in extracellular exosome.<br>[Alliance of Genome Resources, Apr 2022] (**) (§)		
**NLRP14**<br><br>NLR Family Pyrin Domain Containing 14	2,13	0,019	The protein encoded by this gene belongs to the NALP protein family. Members of the NALP protein family typically contain a NACHT domain, a NACHT-associated domain (NAD), a C-terminal leucine-rich repeat (LRR) region, and an N-terminal pyrin domain (PYD). This protein may play a regulatory role in the innate immune system as similar family members belong to the signal-induced multiprotein complex, the inflammasome, that activates the pro-inflammatory caspases, caspase-1 and caspase-5. [RefSeq, Jul 2008] (**)	Germ-cell specific inflammasome component NLRP14 negatively regulates cytosolic nucleic acid sensing to promote fertilization	[81]
				Polyadenylation of in vitro preovulatory- aged oocytes was also increased, along with transcript level declines of Trim28, Nlrp2, Nlrp14 and Zar1.	[82]
**NOS2**<br><br>Nitric Oxide Synthase	1,29	0,001	Nitric oxide is a reactive free radical which acts as a biologic mediator in several processes, such as neurotransmission and antimicrobial and antitumoral activities. This gene encodes a nitric oxide synthase which is expressed in liver and is inducible by a combination of lipopolysaccharide and certain cytokines. Three related pseudogenes are located within the Smith-Magenis syndrome region on chromosome 17.<br>[RefSeq, Jul 2008] (**) (§)	It is involved in the hypoxia stress response in human CCs	[83]
				The increase in iNOS mRNAs expression in CCs is a negative index of oocyte fertilizability and might be an useful tool for oocyte selection	[84]
				NOS2, classified as antioxidants and anti apoptotic genes, was upregulated in heat-stressed bovine GCs	[85]
				Inducible nitric oxide synthase-derived nitric oxide regulates germinal vesicle breakdown and first polar body emission in the mouse oocyte	[86]
				It regulates: folliculogenesis and granulosa cell apoptosis; ovarian steroidogenesis; oocyte maturation; ovulatory response; intercellular commu- nication within ovarian follicle	[87]
**PAX6**<br><br>Paired box 6	1,28	0,019	This gene encodes paired box protein Pax-6, one of many human homologs of the Drosophila melanogaster gene prd. In addition to a conserved paired box domain, a hallmark feature of this gene family, the encoded protein also contains a homeobox domain. Both domains are known to bind DNA and function as regulators of gene transcription. Activity of this protein is key in the development of neural tissues, particularly the eye. This gene is regulated by multiple enhancers located up to hundreds of kilobases distant from this locus. Mutations in this gene or in the		

			enhancer regions can cause ocular disorders such as aniridia and Peter's anomaly. Use of alternate promoters and alternative splicing results in multiple transcript variants encoding different isoforms. Interestingly, inclusion of a particular alternate coding exon has been shown to increase the length of the paired box domain and alter its DNA binding specificity. Consequently, isoforms that carry the shorter paired box domain regulate a different set of genes compared to the isoforms carrying the longer paired box domain. [provided by RefSeq, Mar 2019] (**)		
**PDE4B**<br><br>Phosphodiesterase 4B	1,49	0,013	This gene is a member of the type IV, cyclic AMP (cAMP)-specific, cyclic nucleotide phosphodiesterase (PDE) family. The encoded protein regulates the cellular concentrations of cyclic nucleotides and thereby play a role in signal transduction. Altered activity of this protein has been associated with schizophrenia and bipolar affective disorder. Alternative splicing and the use of alternative promoters results in multiple transcript variants encoding different isoforms.<br>[provided by RefSeq, Jul 2014] (**)		
**PEAR1**<br><br>Platelet Endothelial Aggregation Receptor 1	1,67	0,014	PEAR1 is a platelet receptor that signals upon the formation of platelet-platelet contacts independent of platelet activation and secondary to platelet aggregation ([88] [OMIM, Mar 2008] (**)		
**PLA2G4D**<br><br>Phospholipase A2 Group IVD	1,73	0,030	The phospholipase A2 enzyme family, including PLA2G4D, catalyze the hydrolysis of glycerophospholipids at the sn-2 position and then liberate free fatty acids and lysophospholipids [89] [OMIM, Jun 2009] (*) (§)	It can directly regulate ovarian follicle development, or indirectly influence leptin secretion involved in the regulation of the reproductive endocrine and physiological systems during the anestrous season.	[90]
				First report of transcriptomes of gonads of hybrid triploid loaches and their parents, providing a deep insight into the molecular mechanism controlling the fertility of hybrid triploid fish. Following PPI network analyses, 54 differentially expressed genes, including PLCB4, cyp17a1 and Pla2g4d, were mined, yielding candidate genes involved in the poor fertility of hybrid triploid loaches	[91]
**RDH5**<br><br>Retinol Dehydrogenase 5	1,36	0,012	This gene encodes an enzyme belonging to the short-chain dehydrogenases/reductases (SDR) family. This retinol dehydrogenase functions to catalyze the final step in the biosynthesis of 11-cis retinaldehyde, which is the universal chromophore of visual pigments. Mutations in this gene cause autosomal recessive fundus albipunctatus, a rare form of night blindness that is characterized by a delay in the regeneration of cone and rod photopigments. [RefSeq, Dec 2010] (**)		
**RSAD2**<br><br>Radical S-Adenosyl Methionine Domain Containing 2	1,46	0,015	The protein encoded by this gene is an interferon-inducible antiviral protein that belongs to the S-adenosyl-L-methionine (SAM) superfamily of enzymes. The protein plays a role in cellular antiviral response and innate immune signaling. Antiviral effects result from inhibition of viral RNA replication, interference in the secretory pathway, binding to viral proteins and dysregulation of cellular lipid metabolism. The protein has been found to inhibit both DNA and RNA viruses, including influenza virus, human immunodeficiency virus (HIV-1) and Zika virus. [RefSeq, Sep 2020] (**)	Role in neurotensin expression, regulation, and function during the ovulatory period in the Mouse Ovary	[92]
**SERTAD4**<br><br>SERTA Domain Containing 4	1,27	0,033	Predicted to be located in nucleus.<br>[provided by Alliance of Genome Resources, Apr 2022] (**)		
**SLC3A1**<br><br>Solute Carrier Family 3 Member 1	1,69	0,016	This gene encodes a type II membrane glycoprotein which is one of the components of the renal amino acid transporter which transports neutral and basic amino acids in the renal tubule and intestinal tract. Mutations and deletions in this gene are associated with cystinuria. [RefSeq, Jul 2008] (**)		
**SLC6A15**<br><br>Solute Carrier Family	1,37	0,003	This gene encodes a member of the solute carrier family 6 protein family which transports neutral amino acids. The encoded protein is thought to play a role in		

6 Member 15			neuronal amino acid transport and may be associated with major depression. [RefSeq, Feb 2012] (**)		
**SLC24A5**<br><br>Solute Carrier Family 24 Member 5	1,68	0,034	This gene is a member of the potassium-dependent sodium/calcium exchanger family and encodes an intracellular membrane protein with 2 large hydrophilic loops and 2 sets of multiple transmembrane-spanning segments. Sequence variation in this gene has been associated with differences in skin pigmentation.<br>[RefSeq, Jul 2008] (**)		
**SLC27A3**<br><br>Solute Carrier Family 27 Member 3	1,29	0,008	This gene belongs to a family of integral membrane proteins and encodes a protein that is involved in lipid metabolism. The increased expression of this gene in human neural stem cells derived from induced pluripotent stem cells suggests that it plays an important role in early brain development. Naturally occurring mutations in this gene are associated with autism spectrum disorders. [RefSeq,Feb2017] (**) (§)	It is involved in energetic homeostasis modifications in response to heat stress in GCs	[85]
**SPTLC3**<br><br>Serine palmitoyltransferase Long Chain Base Subunit 3	1,43	0,007	This gene encodes a subunit of the serine palmitoyltransferase complex which catalyzes the rate-limiting step in sphingolipid biosynthesis. This subunit metabolizes lauroyl- and myristoyl-CoA and generates C14 and C16-sphingoid bases.<br>[RefSeq, Mar 2017] (**)		
**TRAM1L1**<br><br>Translocation Associated Membrane Protein 1 Like 1	1,44	0,019	Predicted to be involved in protein insertion into ER membrane. Predicted to be integral component of endoplasmic reticulum membrane.<br>[Alliance of Genome Res., Apr 2022] (**)		
**YBX2**<br><br>Y-box binding protein 2	1,96	0,014	This gene encodes a nucleic acid binding protein which is highly expressed in germ cells. The encoded protein binds to a Y-box element in the promoters of certain genes but also binds to mRNA transcribed from these genes. Pseudogenes for this gene are located on chromosome 10 and 15.<br>[RefSeq, Feb 2012] (**) (§)	Three genes (mitochondrial translational initiation factor 3 (IF3), heat shock transcription factor 5 (HSF5) and Y box binding protein 2 (YBX2)) were differentially expressed in oocytes, with all being more abundant in oocytes from young mares. The results of the present study confirm there are age-related differences in gene expression in equine COCs, which may be associated with the lower quality and decreased developmental competence of oocytes from aged mares.	[93]
lincRNA	1,72	0,002			
lincRNA	2,01	0,018			
lincRNA	2,05	0,011			
lincRNA	1,96	0,016			
lincRNA	2,02	0,007			
lincRNA	2,41	0,006			
lincRNA	1,91	0,045			
lincRNA	2,15	0,010			
lincRNA	2,69	0,003			
lincRNA	2,48	0,001			
LOC101118398	1,67	0,001			
LOC101122984	2,58	0,016			
LOC101114173	1,83	0,007			
LOC101123627	1,67	0,011			
LOC101120495	1,56	0,001			
LOC443326	1,82	0,040			
LOC443320	1,73	0,028			
miRNA	1,84	0,002			
Mt_tRNA	1,76	0,002			
Mt_tRNA	1,56	0,009			
N	2,60	0,001			
N	1,23	0,001			
N	1,34	0,001			
N	1,64	0,006			
N	1,87	0,009			
N	2,01	0,005			
N	2,21	0,013			

Log2 = Fold Change
(*) RNA expressed in normal human ovary (GTEx)
(**) RNA expressed in normal human ovary (GTEx and Illumina)
(§) Protein expression in the ovary (Moped and Proteomics DB)
