# Supplementary material for: Cumulus Cell Transcriptome after Cumulus-Oocyte Complex Exposure to Nanomolar Cadmium in an In Vitro Animal Model of Prepubertal and Adult Age
Source: Biology (Basel). 2023 Feb 4;12(2):249. doi: 10.3390/biology12020249 (PMC9953098; doi:10.3390/biology12020249)
Supplement: Supplementary file 1 [file biology-12-00249-s001.zip › Table S2 - Martino R2.pdf]

Table S2: Downregulated genes in CCs isolated from adult sheep and exposed in vitro to nanomolar cadmium

Gene symbol and name	Log2	P value	Gene Cards (*) (**) (§)	PubMed data found with key words: Cumulus, Granulosa, Oocyte, Ovary	References
**CIDEC**<br><br>Cell death Inducing DFFA like Effector C	-1,87	0,024	This gene encodes a member of the cell death-inducing DNA fragmentation factor-like effector family. Members of this family play important roles in apoptosis. The encoded protein promotes lipid droplet formation in adipocytes and may mediate adipocyte apoptosis. This gene is regulated by insulin and its expression is positively correlated with insulin sensitivity. Mutations in this gene may contribute to insulin resistant diabetes. [RefSeq, Dec 2010] (**) (§)	Lipid droplet proteins perilipin, perilipin-2, cell death-inducing DNA fragmentation factor 45-like effector CIDE-A and CIDE-B were detected in the mouse ovary by immunohistochemistry, but only perilipin-2 was associated with lipid droplets in the oocyte	[94]
				Differences in the expression of CIDEa and CIDEc in 20 different tissues in broiler chickens were examined. Both CIDEa and CIDEc mRNA transcripts were predominantly but variably expressed in white adipose tissue (WAT) but were also expressed at moderate levels in the kidney and liver and at lower levels in the ovary	[95]
**CNTN3**<br><br>Contactin 3	-1,67	0,019	Predicted to be involved in cell adhesion and nervous system development. Predicted to be located in extracellular region and plasma membrane. Predicted to be active in neuron projection. [Alliance of Genome Resources, Apr 2022] (**)	Associated with the methylation pattern of specific gene loci in infertile pigs	[96]
				Associated with reproduction related body shape traits in bovine	[97]
**CORO1A**<br><br>Coronin 1A	-1,36	0,007	This gene encodes a member of the WD repeat protein family. WD repeats are minimally conserved regions of approximately 40 amino acids typically bracketed by gly-his and trp-asp (GH-WD), which may facilitate formation of heterotrimeric or multiprotein complexes. Members of this family are involved in a variety of cellular processes, including cell cycle progression, signal transduction, apoptosis, and gene regulation. [RefSeq, Sep 2010] (**) (§)		
**CPNE5**<br><br>Copine	-1,21	0,008	Calcium-dependent membrane-binding proteins may regulate molecular events at the interface of the cell membrane and cytoplasm. This gene is one of several genes that encode a calcium-dependent protein containing two N-terminal type II C2 domains and an integrin A domain-like sequence in the C-terminus. [RefSeq, Sep 2015] (**)	SNP detection using RNA-sequences of candidate genes associated with puberty in cattle	[98]
**ISL2**<br><br>ISL LIM Homeobox 2	-1,76	0,010	Enables sequence-specific double-stranded DNA binding activity. Predicted to be involved in axonogenesis; neuron fate specification; and regulation of transcription by RNA polymerase II. Predicted to act upstream of or within negative regulation of neuron differentiation and neuron differentiation. Predicted to be part of chromatin. Predicted to be active in nucleus. [provided by Alliance of Genome Resources, Apr 2022] (*)<br><br>Cloned the full-length cDNA of human Isl-2 from a human embryo heart cDNA library. The gene contains six exons and spans 5.7 kb in chromosome 15q23 region, and transcribes a 1.9 kb mRNA that encodes a protein with 359 amino acid residues. The predicted protein, containing two tandem LIM motifs in N-terminal and a homeodomain domain (LIM-HD), is well conserved, especially in the LIM and DNA-binding domains. LIM-HD proteins are suggested as major transcriptional regulators which, in cooperation with other transcription factors, play critical roles in several developing systems and organs. Northern blot analysis shows that human Isl-2 is expressed in every human tissue examined at adult stage and during embryonic developmental stages from 34 days to 24 weeks at different levels in tissues. The broad expression of Isl-2 gene in tissues during embryogenesis and adult development suggests that it may be involved in both differentiation and maintenance of these tissues and might play an important role. [99]<br><br>The exact function of Isl-2 is unknown, but the conserved LIM and homeodomain contained in its sequence provided insight into its potential role in specific protein–protein interaction during transcription regulation [99].		
**LY6G6C**<br><br>Lymphocyte Antigen	-1,91	0,013	LY6G6C belongs to a cluster of leukocyte antigen-6 (LY6) genes located in the major histocompatibility complex (MHC) class III region on chromosome 6. Members of the LY6 superfamily	Significant increase in levels of Ly6G6C+ cells in the spleen of mice, which correlate with	[101]

6 Family Member G6C			typically contain 70 to 80 amino acids, including 8 to 10 cysteines. Most LY6 proteins are attached to the cell surface by a glycosylphosphatidylinositol (GPI) anchor that is directly involved in signal transduction [100]<br>[OMIM, Mar 2008] (**)	increased anti-HSP90 antibody production. Ovarian- and granulosa-cell populations also showed increased infiltration of leukocytes and neutrophil and monocyte populations, which may have led to ovarian follicular degeneration that manifested as empty follicles.	
**MCP1**<br><br>Also known as CCL2 gene C-C motif chemokine ligand 2	-2,21	0,028	Chemokines are a superfamily of secreted proteins involved in immunoregulatory and inflammatory processes. The superfamily is divided into four subfamilies based on the arrangement of N-terminal cysteine residues of the mature peptide. This chemokine is a member of the CC subfamily which is characterized by two adjacent cysteine residues. This cytokine displays chemotactic activity for monocytes and basophils but not for neutrophils or eosinophils. It has been implicated in the pathogenesis of diseases characterized by monocytic infiltrates, like psoriasis, rheumatoid arthritis and atherosclerosis. It binds to chemokine receptors CCR2 and CCR4. Elevated expression of the encoded protein is associated with severe acute respiratory syndrome coronavirus 2 infection. [RefSeq, Aug 2020] (**)	It is expressed in the follicular wall	[102]
				It participates to inflammatory processes involved in luteolysis of the bovine corpus luteum	[103]
**MYCN**<br><br>MYCN Proto-Oncogene, BHLH Transcription Factor	-1,23	0,047	This gene is a member of the MYC family and encodes a protein with a basic helix-loop-helix (bHLH) domain. This protein is located in the nucleus and must dimerize with another bHLH protein in order to bind DNA. Amplification of this gene is associated with a variety of tumors, most notably neuroblastomas. [RefSeq, Jun 2014] (**)	It is a proto-oncogene associated with cell cycle and oocyte maturation pathways	[104]
**PRSS50**<br><br>Serine protease 50	-1,80	0,001	Enables threonine-type endopeptidase activity. Involved in proteolysis. Located in endoplasmic reticulum. [provided by Alliance of Genome Resources, Apr 2022] (**) (§)		
**PSMA8**<br><br>Protasome 20S Subunit Alpha 8	-1,59	0,044	Predicted to be involved in meiotic cell cycle and proteasomal protein catabolic process. Located in extracellular exosome and nucleus. [provided by Alliance of Genome Resources, Apr 2022] (**) (§)<br>This gene plays a role of the Nsd2-dependent (Nuclear receptor binding SET domain protein 2) chromatin remodeling during spermatogenesis and provide clues to the molecular mechanisms in epigenetic abnormalities impacting male reproductive health [105]		
**RASGRP3**<br><br>RAS Guanly Releasing Protein 3	-1,84	0,008	The protein encoded by this gene is a guanine nucleotide exchange factor that activates the oncogenes HRAS and RAP1A. Defects in this gene have been associated with systemic lupus erythematosus and several cancers.<br>[RefSeq, Mar 2017] (**)		
**RGS4**<br><br>Regulator of G protein Signaling 4	-1,21	0,001	Regulator of G protein signaling (RGS) family members are regulatory molecules that act as GTPase activating proteins (GAPs) for G alpha subunits of heterotrimeric G proteins. RGS proteins are able to deactivate G protein subunits of the Gi alpha, Go alpha and Gq alpha subtypes. They drive G proteins into their inactive GDP-bound forms. Regulator of G protein signaling 4 belongs to this family. All RGS proteins share a conserved 120-amino acid sequence termed the RGS domain. Regulator of G protein signaling 4 protein is 37% identical to RGS1 and 97% identical to rat Rgs4. This protein negatively regulate signaling upstream or at the level of the heterotrimeric G protein and is localized in the cytoplasm.<br>[RefSeq, Jul 2008] (**)	Downregulated in ovarian GCs of infertile women with diminished ovarian reserve	[106]
**SLC12A5**<br><br>Solute Carrier Family 12 Member 5	-1,80	0,018	K-Cl cotransporters are proteins that lower intracellular chloride concentrations below the electrochemical equilibrium potential. The protein encoded by this gene is an integral membrane K-Cl cotransporter that can function in either a net efflux or influx pathway, depending on the chemical concentration gradients of potassium and chloride. The encoded protein can act as a homomultimer, or as a heteromultimer with other K-Cl cotransporters, to maintain chloride homeostasis in neurons.<br>[RefSeq, Sep 2008] (**)	Ovarian overexpression of SLC12A5 is associated with tumor progression and poor survival in women with ovarian carcinoma	[107]
**TARS3**<br><br>Threonyl-tRNA Synthetase 3	-1,22	0,014	Predicted to enable threonine-tRNA ligase activity. Predicted to be involved in threonyl-tRNA aminoacylation. Predicted to be located in cytoplasm and nucleus.<br>[Alliance of Genome Resources, Apr 2022] (**)		
**THEM6**<br><br>Thioesterase Superfamily Member 6	-1,22	0,040	Predicted to be located in extracellular region.<br>[provided by Alliance of Genome Resources, Apr 2022] (**) (§)		
**TSPAN18**	-1,31	0,033	Predicted to be integral component of membrane. Predicted to be integral component of plasma membrane. [Alliance of Genome	Recent studies brought the evidences that some cell	[108]

Tetraspanin 18 also known as CD18 Cluster of Differentiation18			Resources, Apr 2022] (**) (§)	surface molecules associated with immune system (cluster of differentiation (CD) molecules) may be involved in the fertilization process. The experimental observations regarding the function of CD9, CD49f/CD29, CD46 and CD11b/CD18 have led to the construction of general hypothesis of fertilization comprising the interaction of these CD molecules in binding and fusion of sperm and egg. The model for the role of integrin CD11b/CD18 and CD46 in fertilization is based on the interaction of these two molecules through complement C3 fragments which may serve as bridging ligands between sperm CD46 and oocyte CD11b/CD18 and facilitate apposition of the sperm inner acrosomal membrane with oolemma.	
**TTC39B**<br><br>Tetratricopeptide Repeat Domain 39B	-1,43	0,013	Predicted to be involved in several processes, including cholesterol homeostasis; negative regulation of cholesterol storage; and regulation of cholesterol efflux.<br>[Alliance of Genome Resources, Apr 2022] (**) (§)		
**TTC9**<br><br>Tetratricopeptide Repeat Domain 9	-1,29	0,047	This gene encodes a protein that contains three tetratricopeptide repeats. The gene has been shown to be hormonally regulated in breast cancer cells and may play a role in cancer cell invasion and metastasis. [RefSeq, Mar 2009] (**)		
**TXK**<br><br>TXK Tyrosin Kinase	-1,59	0,048	Predicted to enable non-membrane spanning protein tyrosine kinase activity. Involved in positive regulation of interferon-gamma-mediated signaling pathway; positive regulation of macromolecule metabolic process; and protein autophosphorylation. Located in cytoplasm and nucleus. [Alliance of Genome Res, Apr 2022] (**)		
**U6**<br><br>RNA, U6 Small Nuclear 1	-1,71	0,041	RNU6-1 (RNA, U6 Small Nuclear 1) is an RNA Gene, and is affiliated with the snRNA class. Diseases associated with RNU6-1 include Poikiloderma With Neutropenia. Among its related pathways are Spliceosomal Splicing Cycle. (*)	It is expressed in bovine oocytes at all meiotic stages and in preimplantation embryos, with highest expression levels in MII stage.	[109]
**ZMYND12**<br><br>Zinc Finger MYND-type Contaning 12	-1,93	0,015	Predicted to enable metal ion binding activity.<br>[Alliance Genome Res., Apr 2022] (**) (§)		
N	-1,33	0,037			
N	-1,38	0,022			
N	-1,56	0,013			
N	-1,38	0,003			
N	-1,73	0,014			

Log2 = Fold Change
(*) RNA expressed in normal human ovary (GTEx)
(**) RNA expressed in normal human ovary (GTEx and Illumina)
(§) Protein expression in the ovary (Moped and Proteomics DB)
