# Supplementary material for: Cumulus Cell Transcriptome after Cumulus-Oocyte Complex Exposure to Nanomolar Cadmium in an In Vitro Animal Model of Prepubertal and Adult Age
Source: Biology (Basel). 2023 Feb 4;12(2):249. doi: 10.3390/biology12020249 (PMC9953098; doi:10.3390/biology12020249)
Supplement: Supplementary file 1 [file biology-12-00249-s001.zip › Table S3 - Martino R3.pdf]

Table S3: Upregulated genes in CCs isolated from prepubertal lambs and exposed in vitro to nanomolar cadmium

Gene ID and description	Log2	P value	Gene Cards (*) (**) (§)	PubMed data found with key words: Cumulus, Granulosa, Oocyte, Ovary	References
**ASTL**<br><br>Astacin Like Metallo-endopeptidase	2,84	1,8E-02	Predicted to enable aspartic-type peptidase activity; glutamic-type peptidase activity; and metalloendopeptidase activity. Predicted to be involved in several processes, including negative regulation of binding activity of sperm to zona pellucida; positive regulation of protein processing; and prevention of polyspermy. Predicted to be located in cortical granule and plasma membrane. [Alliance Genome Resources, Apr 2022] (*) (§)	ASTL signals were significantly reduced in a dose-dependent manner with the decrease in **CC** layers	[110]
**BMP15**<br><br>Bone Morphogenetic Protein 15	2,37	4,0E-02	This gene encodes a secreted ligand of the TGF-beta (transforming growth factor-beta) superfamily of proteins. Ligands of this family bind various TGF-beta receptors leading to recruitment and activation of SMAD family transcription factors that regulate gene expression. The encoded preproprotein is proteolytically processed to generate subunits of a disulfide-linked homodimer, or alternatively, a heterodimer, with the related protein, growth differentiation factor 9 (GDF9). This protein plays a role in oocyte maturation and follicular development, through activation of granulosa cells. Defects in this gene are the cause of ovarian dysgenesis and are associated with premature ovarian failure. [RefSeq, Aug 2016] (*)	Maternal effect genes encode for proteins and transcripts that accumulate during oogenesis and are necessary to support the development of the preimplantation embryo.	[111]
				Among these, two growth factors, namely growth differentiation factor 9 (GDF9) and bone morphogenetic protein 15 (BMP15), are essential for normal follicular development	[112-114]
				The pivotal role of these factors in folliculogenesis and oocyte development is revealed by the discovery of their involvement in relaying communication between the oocyte and its surrounding somatic cells. Interactions between the developing gametes and neighbouring somatic cells are crucial for fertility because this complex dialogue is essential for the coordinated development of both germ cells and somatic cells	[28, 115]
**CENPU**<br><br>Centromere Protein U	2,57	2,8E-02	The centromere is a specialized chromatin domain, present throughout the cell cycle, that acts as a platform on which the transient assembly of the kinetochore occurs during mitosis. All active centromeres are characterized by the presence of long arrays of nucleosomes in which CENPA (MIM 117139) replaces histone H3 (see MIM 601128). MLF1IP, or CENPU, is an additional factor required for centromere assembly. [OMIM, Mar 2008] (*)		
**CULLIN 2**<br><br>domain containig protein	2,46	3,3E-02	Enables ubiquitin protein ligase binding activity. Predicted to be involved in SCF-dependent proteasomal ubiquitin-dependent protein catabolic process and protein ubiquitination. Predicted to act upstream of or within protein catabolic process. Located in nucleoplasm. Part of Cul2-RING ubiquitin ligase complex [Alliance of Genome Resources, Apr 2022] (*) (**) (§)	The E3 ligase Cullin-2 (Cul2) is involved in modulating Bam ubiquitination, which occurs probably at multiple lysine residues of Bam's C-terminal region. Genetic evidence further supports the notion that Cul2-mediated Bam ubiquitination and turnover are essential for germline development in D. melanogaster	[116]
**DSG2**<br><br>Desmoglein 2	1,38	3,5E-02	This gene encodes a member of the desmoglein family and cadherin cell adhesion molecule superfamily of proteins. Desmogleins are calcium-binding transmembrane glycoprotein components of desmosomes, cell-cell junctions between epithelial, myocardial, and other cell types. The encoded preproprotein is proteolytically processed to generate the mature glycoprotein. Mutations in this gene have been associated with arrhythmogenic right ventricular dysplasia, familial, 10. [RefSeq, Jan 2016] (*) (**) (§)	It is part of a cluster of apoptosis up-regulated genes in GCs from atretic follicles	[117]
				DSG2 is expressed in ovarian surface epithelial cells and in GCs, with a role in the development in the bovine fetal ovary	[118]
				It has been identified as a novel molecular subtypes of high-grade serous ovarian cancer	[119]
**GABRA3**<br><br>Gamma Aminobutyric Acid Type A Receptor Subunit Alpha 3	1,52	3,9E-02	GABA is the major inhibitory neurotransmitter in the mammalian brain where GABRA3 gene acts coding the GABA-A receptors, which are ligand-gated chloride channels. Chloride conductance of these channels can be modulated by agents such as benzodiazepines that bind to the GABA-A receptor. At least 16 distinct subunits of GABA-A receptors have been identified.<br>[provided by RefSeq, Jul 2008] (*)		

**HSPA6**<br><br>Heat Shock Protein Family A (Hsp 70) Member 6	1,22	7,2E-03	Enables enzyme binding activity; heat shock protein binding activity; and unfolded protein binding activity. Involved in cellular response to heat and protein refolding. Located in centriole and cytosol. Colocalizes with COP9 signalosome.<br>[Alliance of Genome Res., Apr 2022] (*) (**)		
**MICAL2**<br><br>Microtubule Associated Monooxygenase, Calponin And LIM Domain Containing 2	2,67	2,4E-02	The protein encoded by this gene is a monooxygenase that enhances depolymerization of F-actin and is therefore involved in cytoskeletal dynamics. The encoded protein is a regulator of the SRF signaling pathway. Increased expression of this gene has been associated with cancer progression and metastasis.<br>[provided by RefSeq, Oct 2016] (*) (**)		
**MT1A**<br><br>Metallothionein 1A	3,12	2,3E-21	This gene is a member of the metallothionein family of genes. Proteins encoded by this gene family are low in molecular weight, are cysteine-rich, lack aromatic residues, and bind divalent heavy metal ions. The conserved cysteine residues co-ordinate metal ions using mercaptide linkages. These proteins act as anti-oxidants, protect against hydroxyl free radicals, are important in homeostatic control of metal in the cell, and play a role in detoxification of heavy metals. Disruption of two metallothionein genes in mouse resulted in defects in protection against heavy metals, oxidative stress, immune reactions, carcinogens, and displayed obesity.<br>[provided by RefSeq, Sep 2017] (*) (**)	Knockdown of Runx1 mRNA by small interfering RNA decreased progesterone secretion and reduced levels of mRNA for Cyp11a1, Hapln1, Mt1a, and Rgc32. The hormonally regulated expression of Runx1 in periovulatory follicles, its involvement in progesterone production, and regulation of preovulatory gene expression suggest important roles of RUNX1 in the periovulatory process.	[80]
**MT2A**<br><br>Metallothionein 2A	1,64	3,3E-06	This gene is a member of the metallothionein family of genes. Proteins encoded by this gene family are low in molecular weight, are cysteine-rich, lack aromatic residues, and bind divalent heavy metal ions, altering the intracellular concentration of heavy metals in the cell. These proteins act as anti-oxidants, protect against hydroxyl free radicals, are important in homeostatic control of metal in the cell, and play a role in detoxification of heavy metals. The encoded protein interacts with the protein encoded by the homeobox containing 1 gene in some cell types, controlling intracellular zinc levels, affecting apoptotic and autophagy pathways. Some polymorphisms in this gene are associated with an increased risk of cancer.<br>[provided by RefSeq, Sep 2017] (*) (**)		
**NACHT**<br><br>domain containing protein	2,62	1,6E-02	The protein encoded by this gene is thought to be a cytosolic protein and predicted to contain a NACHT domain and multiple WD40 repeats. Increased expression of this gene was observed in some prostate cancer cell lines. Knocking down expression of this gene results in decreased androgen receptor protein levels, indicating that this gene may be important in modulating androgen receptor activity. Alternative splicing results in multiple transcript variants encoding different isoforms.<br>[provided by RefSeq, Dec 2016] (*) (**)		
**NLRP5**<br>(also Known as **MATER**)<br><br>NLR Family Pyrin Domain Containing 5	2,45	2,7E-02	The protein encoded by this gene belongs to the NALP protein family. Members of the NALP protein family typically contain a NACHT domain, a NACHT-associated domain (NAD), a C-terminal leucine-rich repeat (LRR) region, and an N-terminal pyrin domain (PYD). Expression of this gene is restricted to the oocyte. A mouse gene that encodes a maternal oocyte protein, similar to this encoded protein, is required for normal	MATER expressions in GCs is related with ovarian aging	[120]
				Expression levels of Nlrp5 and Tle6 in oocytes increased from the germinal vesicle stage to metaphase II stage and then gradually decreased during morula and blastocyst stages.	[121]
				Its dynamics expression during sheep oogenesis is related to the age-related decline of oocyte quality	[122]

			early embryogenesis.<br>[provided by RefSeq, Jul 2008] (*) (**)	Previous work has described how the distribution of mouse Mater is regulated in a manner typical of maternal effect genes: Mater is transcripted only during oogenesis, and the protein is stable until the morula stage and early blastocyst but disappears in the late blastocyst	[123]
				The human MATER gene has also been well characterized and the protein is clearly detectable in oocytes (but the expression profile during human folliculogenesis has yet to be characterized. Therefore, this study set out to investigate the distribution of human MATER protein at different follicular maturation stages.	[124-126]
				A specific distribution profile in human oocytes and in their follicular cells, as well as in specific subcellular domains. The finding that MATER is detectable in the follicular cells of growing follicles and persists in cumulus oophorus cells may indicate that MATER is involved in the specific interaction between this cell type and the oocyte that controls the timing of egg ripening and increases the chance of forming a healthy embryo	[126]
				NLRP5 abundance tended to be 19.8-fold greater in theca and CCs in cows with follicular arrest and reduced fertility	[127]
**SLC30A2**<br><br>Solute Carrier Family 30 Member 2	2,83	1,2E-03	The protein encoded by this **gene** is a zinc transporter that acts as a homodimer. The encoded protein plays a role in secreting zinc into breast milk.<br>[RefSeq, Aug 2015] (*) (**)	CCs regulate zinc homeostasis in the oocyte. CCs regulate the timing of the increase in free intracellular zinc in the oocyte during maturation. This effect of CCs is important because an increase in oocyte zinc is required for completion of meiosis and successful establishment of MII arrest	[128-131]
				Thus, acute regulation of free intracellular zinc is a new process regulated by cumulus cell–oocyte interactions in the follicle.	[132]
**WEE2**<br><br>Oocyte Meiosis Inhibiting Kinase	2,41	3,7E-02	Predicted to enable protein tyrosine kinase activity. Predicted to be involved in several processes, including female pronucleus assembly; negative regulation of oocyte maturation; and regulation of meiosis I. Located in cytosol; nucleoplasm; and plasma membrane.<br>[Alliance of Genome Resources, Apr 2022]<br><br>WEE2 (WEE2 Oocyte Meiosis Inhibiting Kinase) is a Protein Coding gene. Diseases associated with WEE2 include Oocyte Maturation Defect 5 and Female Infertility Due To Oocyte Meiotic Arrest. Among its related pathways are Cell cycle. Gene Ontology (GO) annotations related to this gene include transferase activity, transferring phosphorus-containing groups and protein tyrosine kinase activity. An important paralog of this gene is WEE1. (*) (**)	WEE2 and MYT1 kinases synergistically maintain meiotic arrest in mouse oocytes	[133]
				Interestingly, although the expression of WEE2, a gene involved in the maintenance of meiotic arrest in oocytes increased from the preantral to antral stages in vivo and in vitro (present results), its expression was lower in vitro. This could correspond to the maintenance of meiotic arrest of follicle-enclosed oocytes in vivo, and the earlier acquisition of oocyte competence to resume meiosis in vitro. Indeed, downregulation of WEE2 expression resulting from RNAi injection in vitro or transgenic overexpression of RNAi in vivo, has been reported to result in meiotic resumption in mouse and monkey oocytes	[134-137]
				Novel mutations in WEE2: Expanding the spectrum of mutations responsible for human fertilization failure	[138]
lincRNA	2,18	2,3E-02			
N	1,21	2,9E-02			
N	2,50	3,2E-02			
N	-1,26	4,9E-02			

Log2 = Fold Change
(*) RNA expressed in normal human ovary (GTEx)
(**) RNA expressed in normal human ovary (GTEx and Illumina)
(§) Protein expression in the ovary (Moped and Proteomics DB)
